# Case report: Successful combination therapy with isavuconazole and amphotericin B in treatment of disseminated *Candida tropicalis* infection

**DOI:** 10.3389/fmed.2024.1397539

**Published:** 2024-06-24

**Authors:** Qibei Teng, Xueshi Ye, Bei Wang, Xinyue Zhang, Zhizhi Tao, Xiufeng Yin, Qianqian Yang

**Affiliations:** ^1^Department of Hematology, Sir Run Run Shaw Hospital, School of Medicine, Zhejiang University, Hangzhou, China; ^2^Department of Ultrasound in Medicine, Sir Run Run Shaw Hospital, School of Medicine, Zhejiang University, Hangzhou, China; ^3^Department of Traditional Chinese Medicine, The Children's Hospital, School of Medicine, Zhejiang University, Hangzhou, China; ^4^Department of Radiology, Sir Run Run Shaw Hospital, School of Medicine, Zhejiang University, Hangzhou, China

**Keywords:** disseminated candidiasis, *Candida tropicalis*, antifungal combination therapy, isavuconazole, acute leukemia, metagenomic next generation sequencing

## Abstract

Disseminated candidiasis is a severe complication in patients with hematological malignancies who have undergone chemotherapy or hematopoietic stem cell transplantation. It has a high mortality rate. When disseminated candidiasis caused by *Candida tropicalis* involves either the brain or heart, the prognosis is extremely poor. Traditional methods such as cultures are limited in diagnosing disseminated candidiasis. We describe a case report of a 55-year-old man with acute myeloid leukemia who developed candidemia caused by *Candida tropicalis* after chemotherapy, which disseminated extensively to the heart, brain, skin, liver, spleen and kidneys. In this instance, the patient was rapidly diagnosed with candida infection by metagenomic next generation sequencing, and successfully treated with combination therapy of isavuconazole and amphotericin B. The patient continued with treatment of leukemia while simultaneously receiving antifungal therapy, and both leukemia and disseminated candidiasis were effectively controlled. This case report provides real-world experience for treatment of patients with leukemia complicated by disseminated candidiasis.

## 1 Introduction

Disseminated candidiasis refers to the invasion of Candida spp. into the blood system with further spread to two or more non-adjacent organs. It can be divided into acute disseminated candidiasis and chronic disseminated candidiasis according to the clinical manifestation (1). It is a unique clinical manifestation of invasive candidiasis that occurs almost exclusively in immunocompromised people such as patients suffering from hematological malignancies (HM) (2). The morbidity rate of disseminated candidiasis ranges between 3 and 29% (3). Up to a third of patients with disseminated candidiasis die within 3 months due to fulminant infection, with an overall mortality rate of 74% (4). Among the Candida spp. that can cause disseminated candidiasis, *C. tropicalis* is the most common isolate (5–8). *C. tropicalis* infection is difficult to identify in the early stage, which is associated with significant morbidity and mortality (9).

Once disseminated candidiasis is diagnosed, antifungal therapy should be started immediately. Echinocandin or liposomal amphotericin B (AmB) is often the first choice for initial treatment, and voriconazole or fluconazole can be used as an alternative (4). With the introduction of new antifungal agents, the mortality rate of disseminated candidiasis has decreased to 21% (4). However, the mortality rate of disseminated candidiasis involving the central nervous system (CNS) or endocardium still exceeds 50% (10, 11). Moreover, antifungal resistance in clinically relevant Candida spp. (especially *C. tropicalis*) to azoles continues to increase (9). *C. tropicalis* isolates resistant to echinocandin have also been detected by CHIF-NET (12). Limited clinical data demonstrate that a combination of antifungal therapies can improve response rates (4, 13).

For all we know, this is the first case reporting the successful combination of ISA and AMB in a patient with acute leukemia post-chemotherapy, with disseminated candidiasis involving the endocardium, brain, skin, liver, spleen, and kidneys.

## 2 Case presentation

A 55-year-old man arrived at the clinic complaining of anal pain and a fever of approximately 38.3°C for 3 days. A colonoscopy was performed and showed a huge ulcer (6 cm in diameter) in the rectum. Pathology demonstrated mild, chronic inflammation. Because of evidence of pancytopenia [white blood cells 1.3 × 10^9^/L, hemoglobin 84 g/L, platelets 89 × 10^9^/L, absolute neutrophil count (ANC) 0.33 × 10^9^/L], bone marrow examination was performed and the patient was diagnosed with acute myeloid leukemia (AML). The patient was admitted to the Department of Hematology. After antibiotic treatment with imipenem, the patient’s anal pain was relieved and his temperature returned to normal. Meanwhile, C-reactive protein (CRP) levels decreased from 113.5 mg/L to a normal level. He then received idarubicin combined with cytarabine as induction chemotherapy (days 1–7).

**Figure 1:**
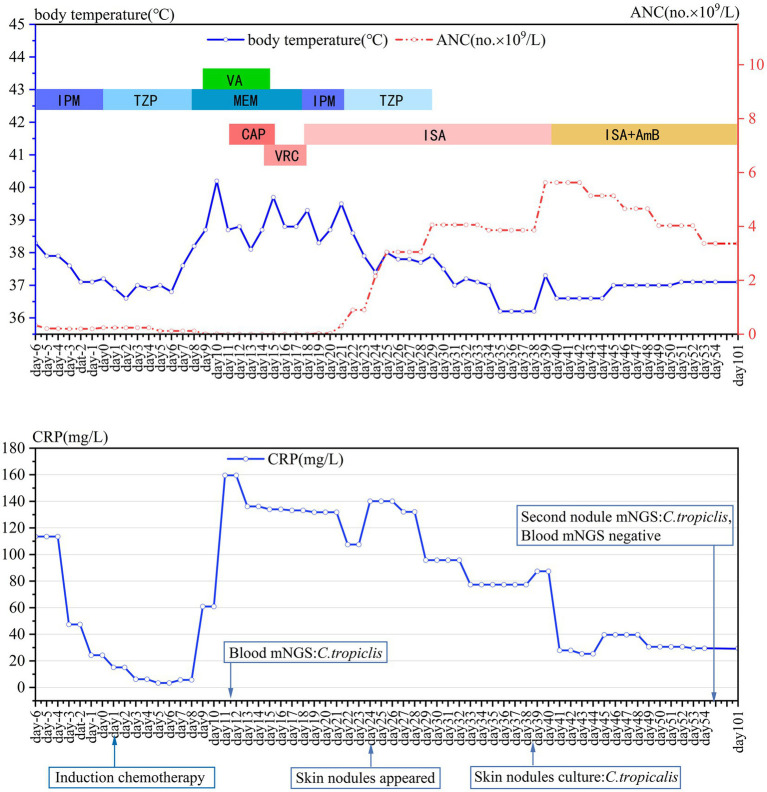
Daily course of the patient’s treatment; curves of body temperature, ANC and CRP. Major events are indicated with arrows. Red dotted line shows ANC in peripheral blood. Blue line on the top shows body-temperature values. Blue line on the bottom shows CRP values. Horizontal color bars show the medications administered: IPM, imipenem; TZP, piperacillin-tazobactam; MEM, meropenem; VA, vancomycin; CAP, caspofungin; VRC, voriconazole; ISA, isavuconazole; AmB, amphotericin B; CRP, C-reactive protein; ANC, the absolute neutrophil count.

After his initial chemotherapy was completed, the patient once again developed a fever of 38.7°C, without any other symptoms, on day 8. Wide-spectrum antibiotics meropenem and vancomycin were given but the fever increased to 40.2°C on day 10. Blood tests showed persistent neutropenia while CRP increased continually (Figure 1). Procalcitonin and (1,3)-β-D-glucan test along with the galactomannan test, all came back negative. A blood sample was tested using metagenomic next generation sequencing (mNGS), and 80 reads of *C. tropicalis* (identification confidence 99%) were reported within 24 h. No other pathogens were detected in the blood sample. Caspofungin (CAS, 70 mg on the first day, followed by 50 mg daily) was used immediately following the result of mNGS on day 11. However, the patient’s condition did not improve obviously and the fever persisted. On day 14, intravenous antimycotic therapy was converted to voriconazole (VCZ, 6 mg/kg q12h on the first day, followed by 4 mg/kg q12h daily). On day 15, the patient developed new symptoms of cough with expectoration, and the high fever persisted. Chest computed tomography (CT) showed multiple, bilateral pulmonary small nodular lesions. A sputum smear was positive for fungi. Test for cytomegalovirus-DNA in blood was negative, and three peripheral blood samples were all culture-negative since the onset of fever. On day 17, we changed the antifungal therapy to isavuconazole (ISA, 200 mg three-times daily for 2 days, then 200 mg once daily thereafter) because of the persistent high fever and elevated alanine transaminase level (113 U/L). Consequently, the patient’s cough with expectoration gradually resolved, and the temperature dropped below 38°C on day 21.

**Figure 2:**
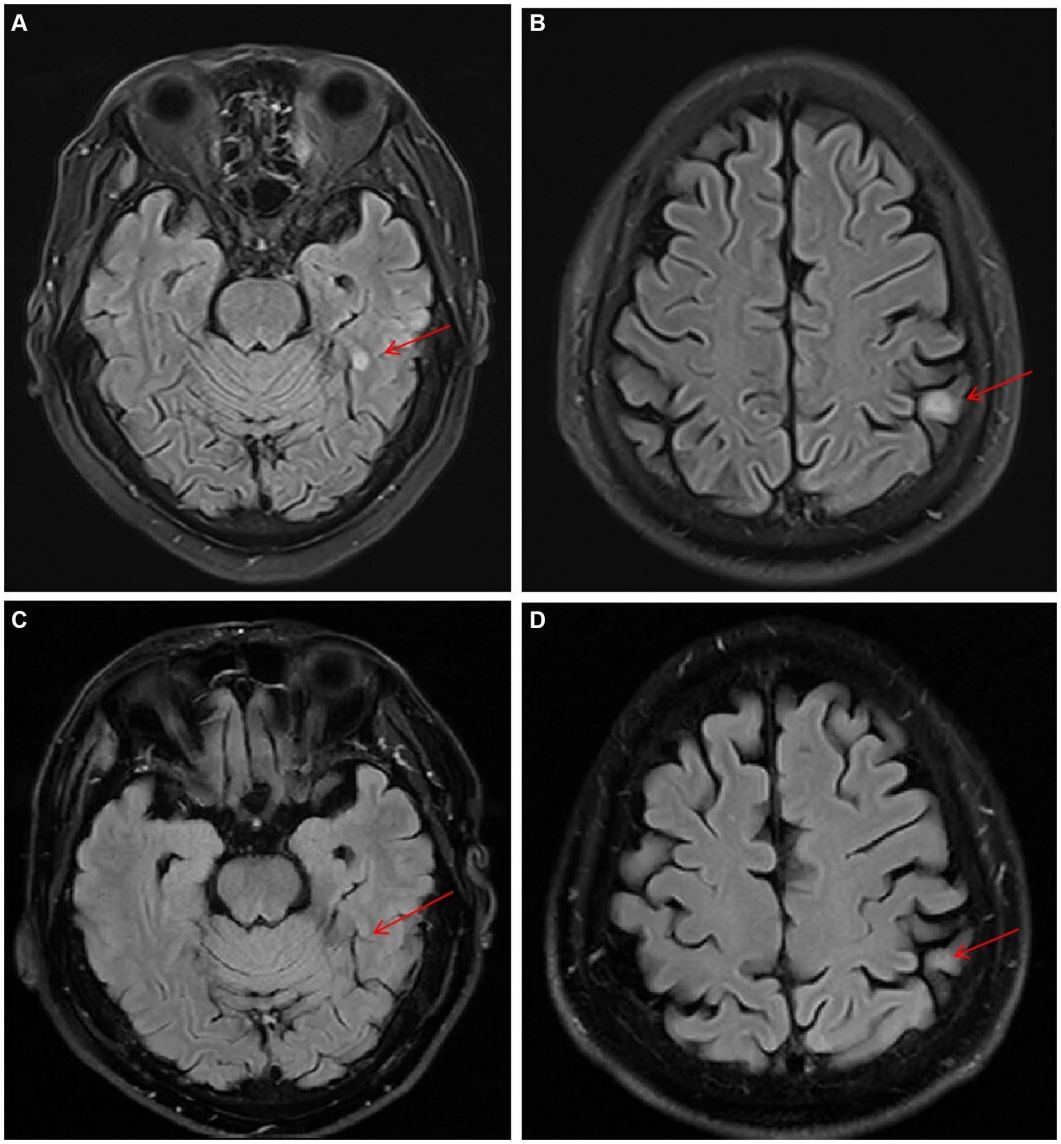
Serial T2-weighted FLAIR MRI images of lesions in the left temporal and parietal lobes (the red arrows). **(A,B)** Day 32. **(C,D)** Day171.

On day 22, neutropenia had resolved (ANC 0.9 × 10^9^/L), and glutamyl transpeptidase was significantly elevated from 69 IU/L to 181 IU/L. The patient found a soybean-sized skin mass under his right eyebrow, a subcutaneous nodule on the left pectoral, and a similar nodule on the neck. A biopsy of the subcutaneous nodule on the left pectoral was performed, and the tissue was sent for culture. On day 25, the patient complained of paroxysmal headache. Brain magnetic resonance imaging (MRI) revealed lesions in both the parietal and left temporal lobes (Figures 2A,B). A lumbar puncture showed normal pressure and cytology within the cerebrospinal fluid (CSF). The level of protein and glucose in CSF was also normal. CSF culture and mNGS were both negative. The patient had a recurrent low fever of between 37.8°C and 37.9°C from day 21 to 29, and subsequently both body temperature and ANC returned to the normal range. Bone marrow aspirate on day 35 showed complete remission of AML.

**Figure 3:**
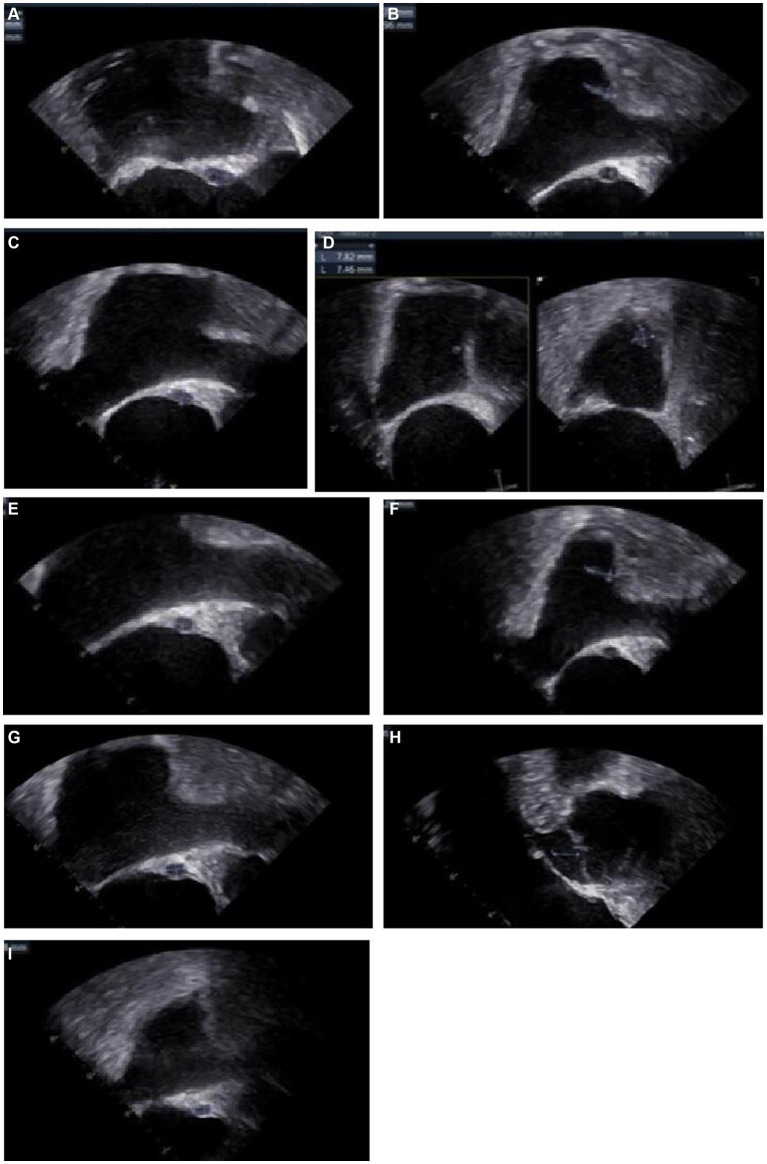
Images of transesophageal echocardiography (TEE) **(A)** Day 43, a fusiform heterogeneous hypoechoic mass (11.5*6.0 mm) in the atrial septum, and a fluid dark area could be seen. **(B)** Day 43, a stream-like echo (11.0*3.7 mm) at the entrance of the superior vena cava. **(C)** Day 55, the mass (10.6*5.7 mm) in the atrial septum. **(D)** Day 55, a moderate echogenic mass (13.0*5.9 mm, basal width 4.6 mm) near the opening of the superior vena cava, with a large amplitude of oscillation during the heart rhythm cycle. A moderate echogenic mass (9.5*7.0 mm, base width 6.9 mm) near the orifice of the inferior vena cava. **(E)** Day 101, the abscess (6.25*4.47 mm) in the atrial septum. **(F)** Day 101, the moderate echoic mass (12.3*3.7 mm, base width 3.4 mm) near the opening of the superior vena cava, the mass near the opening of the inferior vena cava disappeared. **(G)** Day 129, the heterogeneous hypoechoic mass (6.35*4.47 mm) in the atrial septum. **(H)** Day 129, the moderate echogenic mass near the opening of the superior vena cava was not seen. **(I)** Day 235, the heterogeneous hypoechoic mass (6.18*4.07 mm) in the atrial septum.

**Figure 4:**
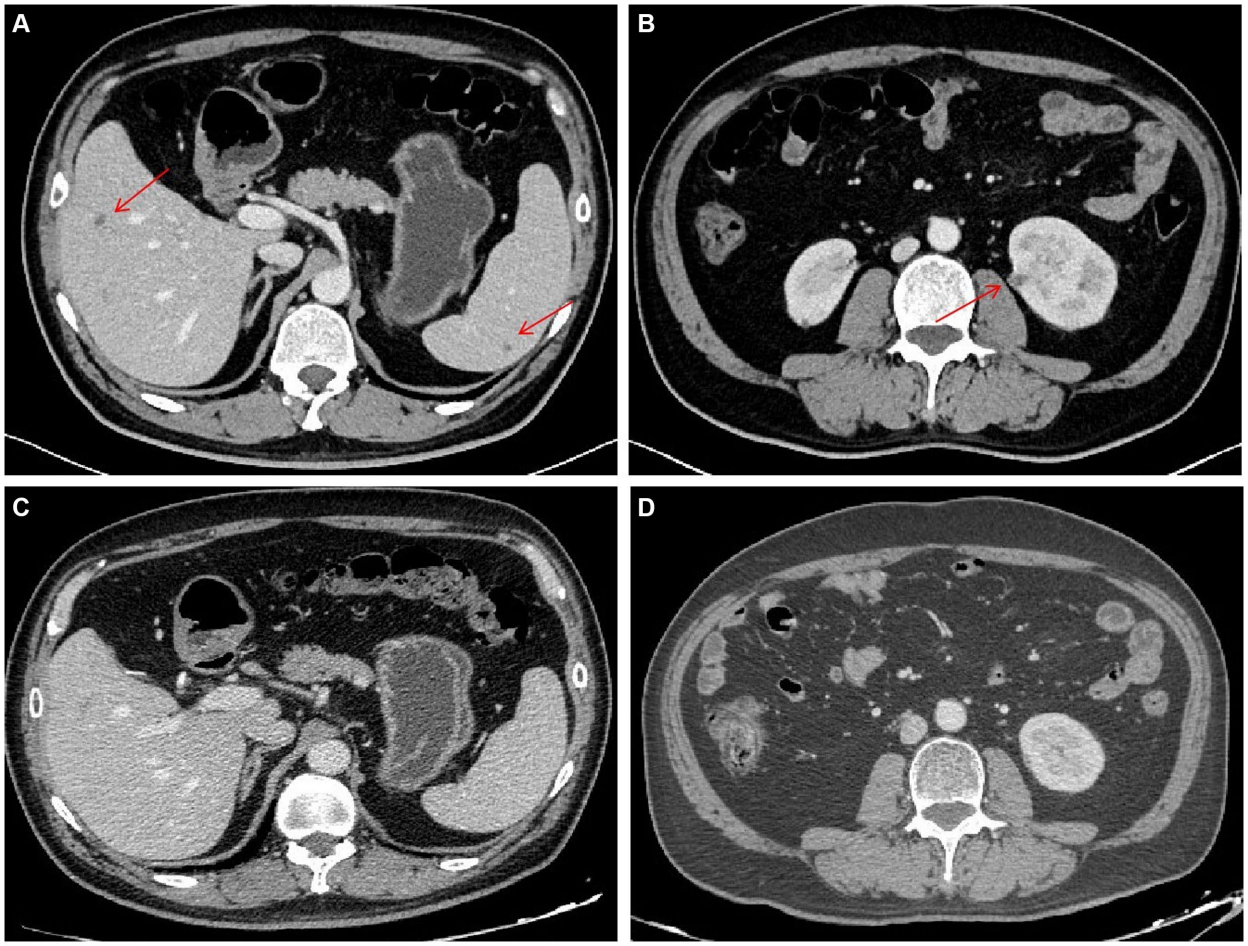
Contrast-enhanced CT images of chronic disseminated candidiasis. **(A,B)** Day 39, multiple small abscesses in the liver, spleen and both kidneys (the red arrows). **(C,D)** Day 171, abscesses almost disappeared.

On day 38, the culture of skin nodule tissue returned positive for *C. tropicalis*, and the antimicrobial susceptibility testing showed AmB susceptible with minimal inhibitory concentration ≤ 0.5 μg/mL (methods and results of identification and susceptibility were in Supplementary material). Disseminated candidiasis was suspected, and thorough physical examinations and imaging studies were performed. The patient reported no fever, headache, or abdominal pain. The nodules on the head and the neck remained the same as before. Cardiac sound was low, along with a murmur in the precordial area. Transesophageal echocardiography (TEE) showed a heterogeneous hypoechoic mass (11.5 × 6 mm) in the atrial septum and an endocardial vegetation in the right atrium (11 × 3.7 mm) (Figures 3A,B). Abdomen contrast-enhanced CT showed multiple small abscesses in the liver, spleen and both kidneys (Figures 4A,B). Chest CT showed bilateral multiple pulmonary small nodules similar to what was observed on day 15. Brain MRI revealed no change in the lesions in the left temporal and parietal lobes.

On day 39, AmB was added to ISA treatment to augment the antifungal effects on the disseminated candidiasis. AmB was given at 5 mg/d on the first day, gradually increased to 30 mg/d, and reduced to 20 mg/d because of elevated creatinine levels from day 81. The patient’s skin nodules started to soften and shrink shortly after the combination therapy had commenced. Follow-up imaging showed the disseminated fungal lesions gradually became smaller. However, a transient enlargement of endocardial vegetation in the right atrium was evident by TEE on day 55 (Figures 3C,D), and several new skin nodules appeared during day 57–64. The biopsy of a new skin nodule was performed. The tissue mNGS still exhibited *C. tropicalis* (287 reads), while the blood mNGS was negative.

The dual antifungal therapy continued for 62 days (from days 39 to 101). All skin nodules eventually disappeared, and lesions in the brain, heart, liver, spleen and kidneys became significantly smaller. Considering stable control of disseminated candidiasis was achieved, ISA was stopped on day 101. Treatment with AmB continued until all lesions had almost disappeared or no longer reduced in size (Figures 3E–I, 2C,D, 4C,D). The cumulative dose of AmB was 2.54 g. Fluconazole (FLU, 400 mg oral once daily) was given as maintenance therapy. During antifungal therapy, creatinine levels had elevated slightly but decreased to normal levels after the dose of AmB was reduced. No other side effects were observed.

The patient was treated with low-dose venetoclax as consolidation therapy for leukemia. The first course of venetoclax initiated on day 65 (100 mg on the first day, 200 mg on the second day, 400 mg from the third day to the seventh day). The second course initiated on day 101 (400 mg for 10 days). The third course initiated on day 140 (400 mg for 14 days). Bone marrow assessment showed that the patient was in complete remission from leukemia.

## 3 Discussion

We describe a complicated case of disseminated candidiasis. This is the first report describing ISA combined with AmB successfully used to treat disseminated candidiasis caused by *C. tropicalis* in a patient with AML. *C. tropicalis* is the main causative agent of disseminated candidiasis in patients with HM (6–8). A study included 35 patients with HM and disseminated candidiasis, with half of Candida spp. identified as *C. tropicalis* (14). Infection caused by *C. tropicalis* is associated with the highest incidence of sepsis and poorest prognosis (15). This phenomenon may be related to more common fluconazole resistance, greater ability to invade damaged gastrointestinal mucosa, and higher virulence of *C. tropicalis* in patients with neutropenia or mucosal infections (16).

In this case report, candidemia caused by *C. tropicalis* developed in a patient with AML after chemotherapy, followed by disseminated candidiasis which affected his endocardium, brain, skin, liver, spleen and kidneys. This case was extremely challenging to treat. The mortality rate of Candida-associated endocarditis can be as high as 50% (10). The optimum therapy for endocarditis in adults is a combination of valve replacement and a long course of antifungal therapy (4). However, this patient could not tolerate cardiac surgery because of his AML. The recommended initial therapy for Candida-associated endocarditis is liposomal AmB, with or without flucytosine, or high-dose echinocandin (4). However, the patient had a poor response to echinocandin in the early stages of candidemia. This patient also had CNS candidiasis. A retrospective study reported by Chaussade et al. showed that the mortality rate associated with CNS candidiasis was 42%, while it was 53% in cases of disseminated infection, and 70% for hematologic malignancies (11). For CNS candidiasis, initial treatment with liposomal AmB with or without oral flucytosine is recommended. However, AmB displays limited CNS penetration, and cannot easily pass through the meninges (17). Flucytosine has good CNS penetration but its toxicity to leukocytes and platelets aggravates bone marrow suppression (18).

Isavuconazole is a new extended-spectrum triazole, has favorable tolerability, fewer nephrotoxic effects, and a similar efficacy against the great mass of Candida spp. when compared with other azoles (4, 19). ISA has shown good blood–brain barrier penetration in animal models, and brain concentration/plasma concentration ratios have been known to approximate 1.8:1 (20, 21). In the retrospective study reported by Schwartz et al., among 36 patients with CNS invasive fungal disease (IFD) (in which 17 patients had hematologic malignancies) treated with ISA, 58.3% of patients achieved a complete or partial clinical response (17). Naeem et al. reported a case of refractory pediatric coccidioidal meningitis with disease stabilization and improvement for 21 months on ISA after treatment with voriconazole and AmB failed (22). These results suggest that ISA has great therapeutic potential in the treatment of IFD involving the CNS. Data related to Candida-associated endocarditis treated with ISA are limited. Two cases have been reported in which ISA was employed for chronic life-long suppression for endocarditis caused by *C. albicans* and *C. tropicalis* (23, 24).

In our case, after diagnosis of candidemia, the initial antifungal therapy using caspofungin followed by voriconazole showed no significant improvement in temperature or clinical symptoms. After treatment with ISA, the patient’s body temperature dropped to normal, and his cough alleviated. However, subsequent systemic imaging showed candidiasis had disseminated to the heart, brain, and many organs. Soon we added AmB in combination with ISA. After 9 weeks of dual antifungal therapy, all lesions were significantly reduced without new or enlarged lesions. The combination therapy of ISA and AmB showed excellent efficacy in treating disseminated candidiasis involving the CNS and endocardium.

The high mortality rate of IFD in immuno-compromised patients has led to new attempts to combine therapy with new antifungal drugs, especially ISA. Odysseos et al. reported a case of refractory disseminated candidiasis in a liver transplant recipient, treated successfully with a dual antifungal therapy of ISA and AmB (13). Cases of disseminated mucormycosis in children with leukemia, treated successfully with ISA and liposomal AmB, have been reported (25, 26). Feys et al. reported a case of disseminated aspergillosis involving CNS and lung, successfully treated by intracerebral liposomal AmB combined with intravenous ISA (27). Furthermore, there have been cases of fungal endocarditis successfully treated with dual antifungal therapy using liposomal AmB and ISA (28, 29). The synergistic effects between different antifungal agents may provide a wider range of antifungal activities. Combination therapy can reduce the dose of drug and thus reduce associated toxicity. In this case, only a slight increase in creatinine was observed after combination therapy, which was promptly reversed by lowering the dose of AmB. No long-term adverse reactions occurred. However, the efficacy and safety of combination of antifungal drugs need more studies in other patient groups.

In addition to drug combinations, another key point for successful treatment is rapid diagnosis. The poor prognosis and high mortality rate of candidemia is partly due to the difficulty in diagnosing in the early stage of disease (9). Blood cultures are very limited in diagnosing invasive candidiasis because of low sensitivity and slow turnaround time (30). The positivity rate of blood cultures has been reported as being as low as 32% in the acute candidemia stage and only 20% in the chronic and disseminated stage (31). New diagnostic methods are needed to complement cultures.

mNGS is a faster methodology of pathogen detection through sequencing of extracted DNA from the sample directly. mNGS has been widely used in the detection of pathogens in infectious diseases including fungal diseases because of its advantages of high throughput and high sensitivity (32, 33). But in the field of candidiasis, the role of mNGS has been poorly studied. Yanqi Jin et al. (34) reported the first case of rapid diagnosis of culture-negative disseminated candidiasis by mNGS of liver biopsy samples. Luo et al. (35) reported three cases of culture-negative disseminated candidiasis diagnosed by mNGS. In our case, the blood culture was negative, while mNGS of the blood sample identified *C. tropicalis* within 24 h, which prompted rapid commencement of appropriate antifungal treatment. When the nodular lesions of the skin recurred, mNGS of a skin biopsy sample quickly confirmed that the skin lesions were caused by *C. tropicalis*, not related to leukemia. The negative mNGS results for the blood sample after antifungal treatment provided us with reliable evidence in evaluating the efficacy of treatment for candidemia. Our experience suggests that mNGS has great potential for the rapid and sensitive diagnosis of candidemia and disseminated candidiasis, and it is a helpful monitoring tool for evaluating the effectiveness of antifungal therapy. However, due to the small number of cases, more studies are needed to confirm the above viewpoints.

23 weeks after candidemia was diagnosed and antifungal therapy was administered, all lesions (except in the heart) gradually became smaller and disappeared, with only calcifications apparent on imaging. In contrast, the endocardial lesions were still present but getting smaller after 32 weeks of treatment. This suggests that Candida-associated endocarditis should be treated for longer periods of time and is consistent with guidelines, which recommend antifungal treatment beyond 2 years for patients who cannot undergo surgical treatment or valve replacement (4, 36).

We also observed that while the combination of ISA and AmB was effective in treatment of disseminated candidiasis, recurrence of lesions can occur in the early course of treatment. This phenomenon was also described by Luo et al. (35), and it does not represent treatment failure or drug resistance. However, it may be related to the recovery of neutrophils and immune reconstitution inflammatory response syndrome (3, 37, 38). This indicates the importance of adhering to and persisting with treatment despite early recurrence of lesions in disseminated candidiasis.

In our case report, chemotherapy was suspended for 9 weeks after induction chemotherapy. After stable control of disseminated candidiasis, we chose the low-dose targeted agent venetoclax to treat leukemia, in addition to the antifungal therapy. AML remained in complete remission and allogeneic hematopoietic stem cell transplantation was planned for long-term survival. This case proves the efficacy and safety of venetoclax combined with antifungal therapy in patients with AML complicated by disseminated candidiasis. Furthermore, ISA is a mild/moderate CYP3A4 inhibitor with fewer drug interactions compared with posaconazole and voriconazole (39), which is safer for leukemia patients who may require targeted drugs such as venetoclax.

## 4 Conclusion

Disseminated candidiasis involving the CNS and endocardium, caused by *C. tropicalis*, is a very challenging problem for clinicians. This report shows that combination antifungal therapy using ISA and AmB is effective in treatment of candidemia and widespread disseminated candidiasis caused by *C. tropicalis* in patients with acute leukemia. The side effects are favorable. These findings suggest that dual antifungal therapy including a novel azole such as ISA provides new options for the treatment of invasive fungal diseases in immunocompromised populations. Finally, as a sensitive etiological diagnostic method, mNGS has great potential in the rapid diagnosis of disseminated candidiasis. More studies are needed to validate new antifungal therapies and etiological detection methods.

## Data availability statement

The raw data supporting the conclusions of this article will be made available by the authors, without undue reservation.

## Ethics statement

The requirement of ethical approval was waived by Clinical Research Ethics Committee of Sir Run Run Shaw Hospital (SRRSH), Zhejiang University School of Medicine for the studies on humans because this case report is a retrospective study, it does not involve additional interventions for the patients. The studies were conducted in accordance with the local legislation and institutional requirements. Written informed consent for participation was not required from the participants or the participants’ legal guardians/next of kin in accordance with the national legislation and institutional requirements. The human samples used in this study were acquired from a by-product of routine care or industry. Written informed consent was obtained from the individual(s) for the publication of any potentially identifiable images or data included in this article. Written informed consent was obtained from the participant/patient(s) for the publication of this case report.

## Author contributions

QT: Data curation, Formal analysis, Software, Writing – original draft, Writing – review & editing. XuY: Conceptualization, Funding acquisition, Methodology, Project administration, Resources, Supervision, Validation, Visualization, Writing – review & editing. BW: Software, Supervision, Visualization, Writing – review & editing. XZ: Conceptualization, Investigation, Software, Writing – review & editing. ZT: Data curation, Methodology, Visualization, Writing – review & editing. XiY: Data curation, Methodology, Software, Writing – review & editing. QY: Project administration, Software, Supervision, Writing – review & editing.
