# Supplementary figures and images for: Case report: Successful combination therapy with isavuconazole and amphotericin B in treatment of disseminated Candida tropicalis infection
Source: Front Med (Lausanne). 2024 Jun 24;11:1397539. doi: 10.3389/fmed.2024.1397539 (PMC11228301; doi:10.3389/fmed.2024.1397539)

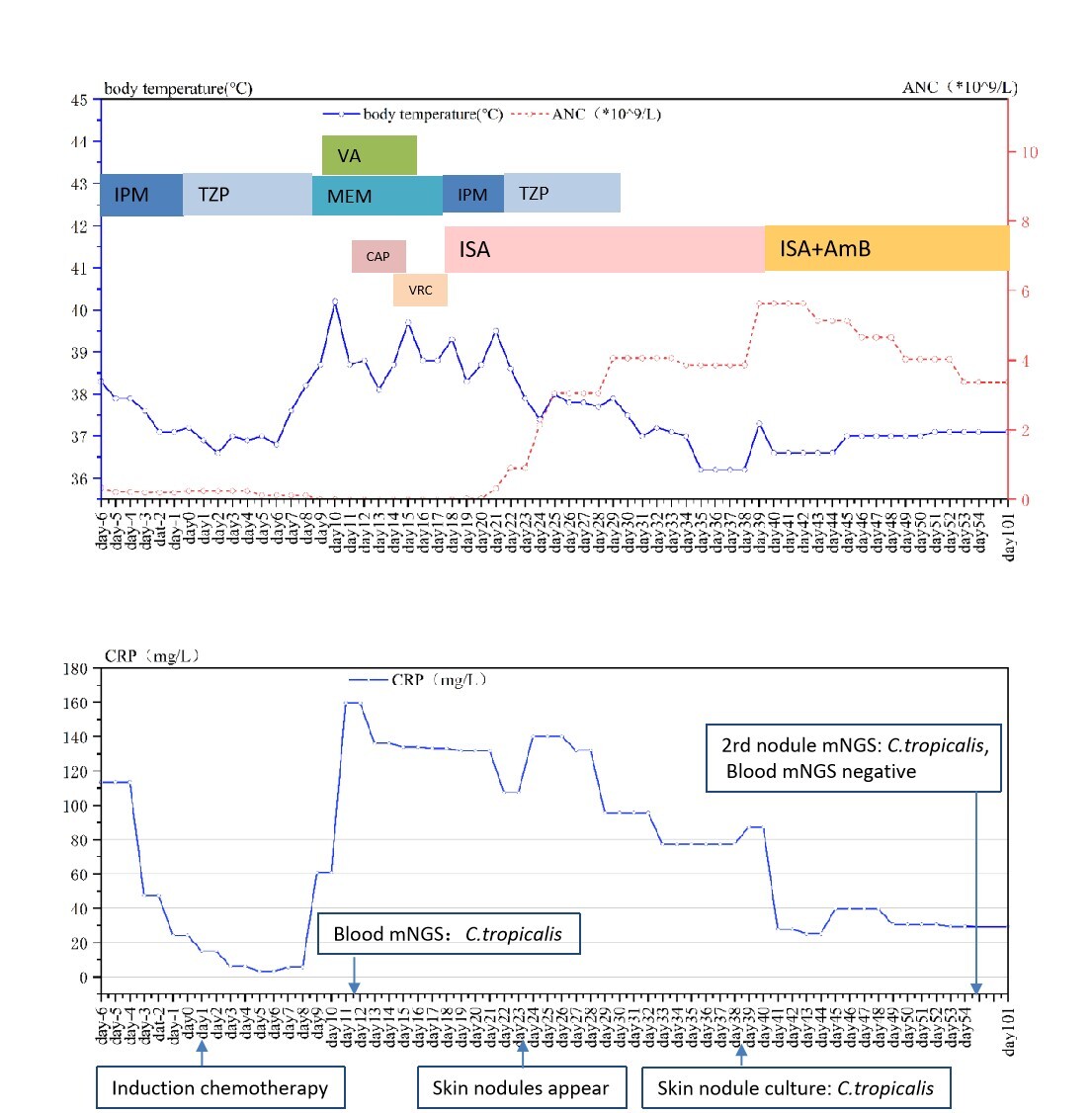

Supplement: Supplementary file 1 [file Data_Sheet_1.ZIP › figure/figure1.jpg]

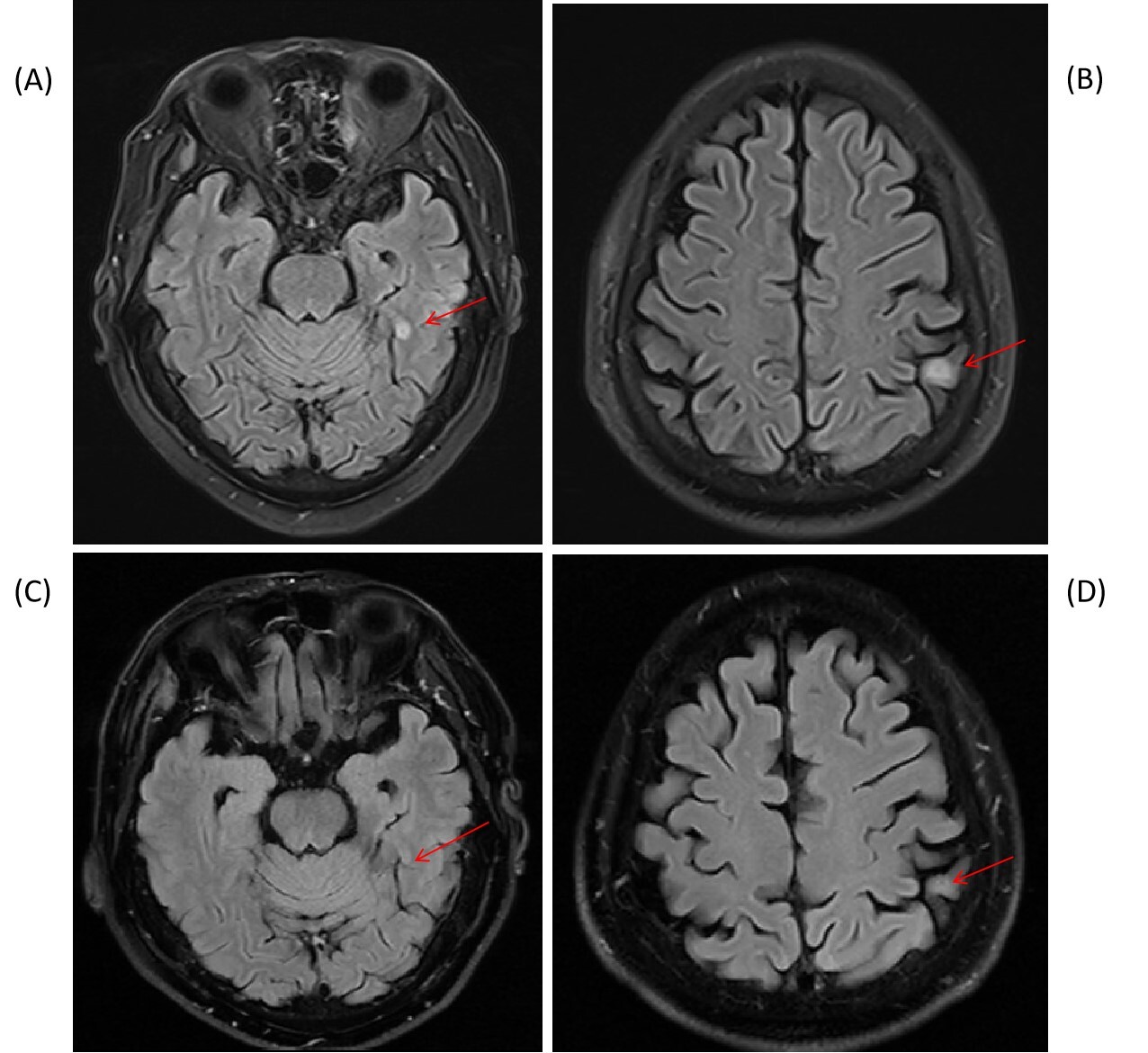

Supplement: Supplementary file 1 [file Data_Sheet_1.ZIP › figure/figure2.jpg]

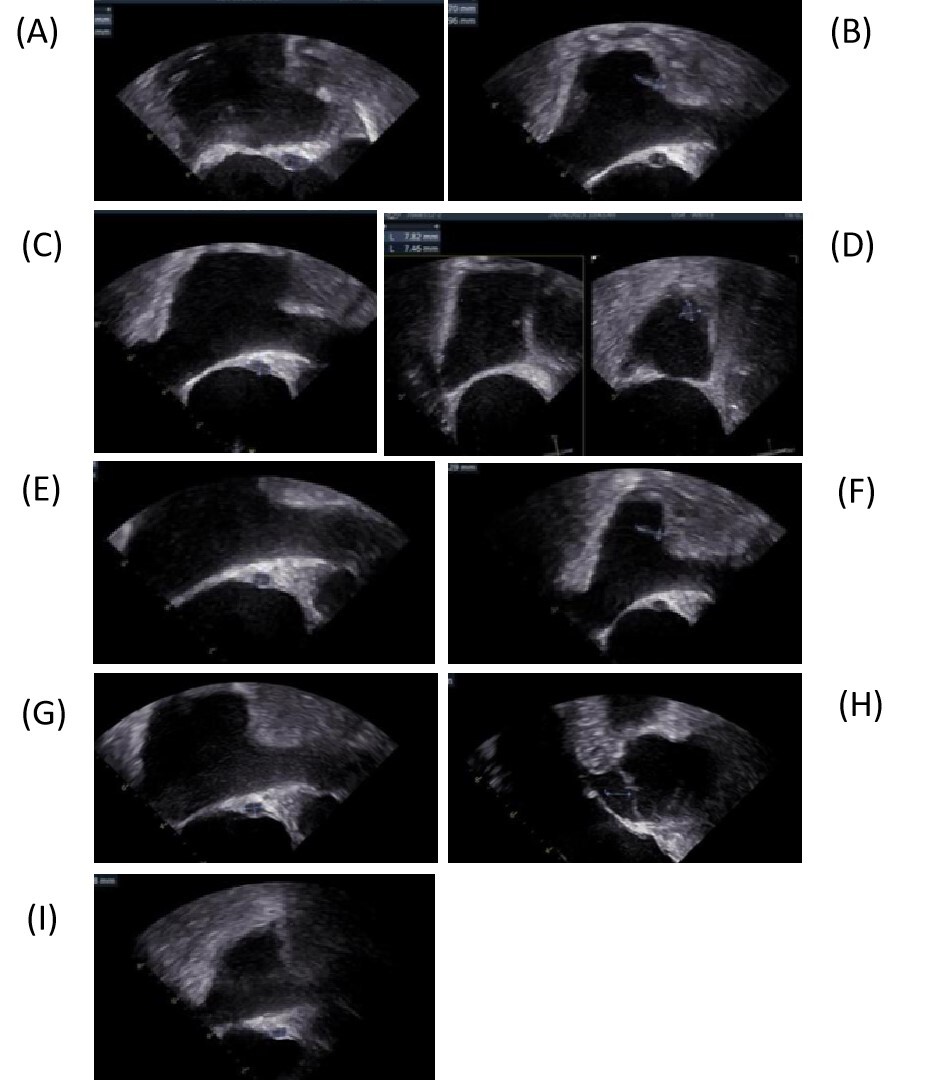

Supplement: Supplementary file 1 [file Data_Sheet_1.ZIP › figure/figure3.jpg]

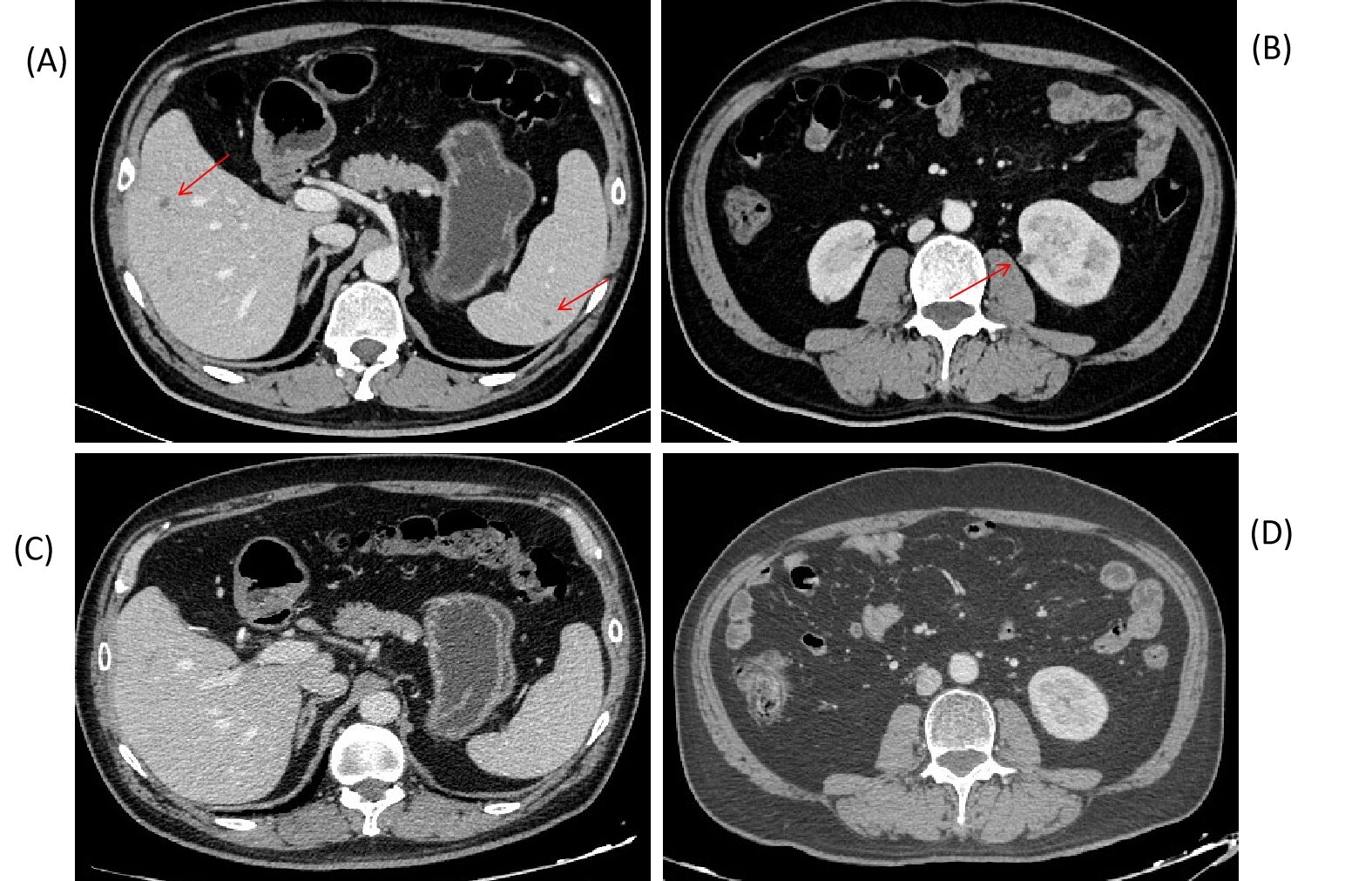

Supplement: Supplementary file 1 [file Data_Sheet_1.ZIP › figure/figure4.jpg]
