# Supplementary material for: Case report: Successful combination therapy with isavuconazole and amphotericin B in treatment of disseminated Candida tropicalis infection
Source: Front Med (Lausanne). 2024 Jun 24;11:1397539. doi: 10.3389/fmed.2024.1397539 (PMC11228301; doi:10.3389/fmed.2024.1397539)
Supplement: Supplementary file 2 [file Data_Sheet_2.docx]

Supplementary Material

Case Report: Successful combination therapy with isavuconazole and amphotericin B in treatment of disseminated *Candida tropicalis* infection

Qibei Teng^1^, Xueshi Ye^1*^, Bei Wang^2^, Xinyue Zhang^3^, Zhizhi Tao^4^, Xiufeng Yin^1^, Qianqian Yang^1^

*** Correspondence:** Xueshi Ye: [Yexueshi2008@zju.edu.cn](mailto:Yexueshi2008@zju.edu.cn)

# The method of nucleic acid extraction, library preparation and sequencing:

The QIAGEN QIAamp® UCP Pathogen DNA Kit was used for DNA nucleic acid extraction. The Illumina Nextera XT DNA Library Prep Kit was used to construct the library for DNA and cDNA samples. Libraries were loaded onto an Illumina Nextseq CN500 sequencer for sequencing. Sequences with low sequencing quality, adaptor sequences, repetitive sequences, and sequences less than 50bp were removed by the software Trimmomatic. Identification and exclusion of human sequence data were performed by mapping to the human reference genome (hg38) using Burrows-Wheeler Aligner. Non-host (human) sequences were aligned with representative genome databases of microorganisms using SNAPv1.0beta.18.
