# Supplementary material for: Case report: Successful combination therapy with isavuconazole and amphotericin B in treatment of disseminated Candida tropicalis infection
Source: Front Med (Lausanne). 2024 Jun 24;11:1397539. doi: 10.3389/fmed.2024.1397539 (PMC11228301; doi:10.3389/fmed.2024.1397539)
Supplement: Supplementary file 3 [file Data_Sheet_3.docx]

Supplementary Material

Case Report: Successful combination therapy with isavuconazole and amphotericin B in treatment of disseminated *Candida tropicalis* infection

Qibei Teng^1^, Xueshi Ye^1*^, Bei Wang^2^, Xinyue Zhang^3^, Zhizhi Tao^4^, Xiufeng Yin^1^, Qianqian Yang^1^

*** Correspondence:** Xueshi Ye: Yexueshi2008@zju.edu.cn

# The identification methods of Candida tropicalis isolated from skin nodule tissue

Species identification was completed by using Matrix-assisted laser desorption ionization-time-of-flight (MALDI-TOF, bioMérieux, France).

# The methods and results of the antimicrobial susceptibility testing (AST)

AST was performed by ATB FUNGUS 3 (bioMérieux, France) and the result was interpreted according to the guideline of CLSI M57 for fluconazole and voriconazole. Here is the result of AST.

| Antifungal agents | Susceptibility | Minimal inhibitory concentration(μg/ml) |
| --- | --- | --- |
| 5-fluorocytosine | Not applicable | ≤4 |
| Amphotericin B | Susceptible | ≤0.5 |
| Fluconazole | Not applicable | ≤1 |
| Itraconazole | Not applicable | ≤0.125 |
| Voriconazole | Susceptible | ≤0.06 |
