# Supplementary material for: Case report: Successful combination therapy with isavuconazole and amphotericin B in treatment of disseminated Candida tropicalis infection
Source: Front Med (Lausanne). 2024 Jun 24;11:1397539. doi: 10.3389/fmed.2024.1397539 (PMC11228301; doi:10.3389/fmed.2024.1397539)
Supplement: Supplementary file 4 [file Data_Sheet_4.docx]

Supplementary Material

Case Report: Successful combination therapy with isavuconazole and amphotericin B in treatment of disseminated *Candida tropicalis* infection

Qibei Teng^1^, Xueshi Ye^1*^, Bei Wang^2^, Xinyue Zhang^3^, Zhizhi Tao^4^, Xiufeng Yin^1^, Qianqian Yang^1^

*** Correspondence:** Xueshi Ye: Yexueshi2008@zju.edu.cn

# mNGS sequence of C. tropicalis from skin tissue

>NDX551268_RUO:41:HNJG3BGXN:4:23611:17483:9576

TTGCCCAGACTAGGATTTGTGCTCTCTTACGCTTTAATTCCATATATCAT

>NDX551268_RUO:41:HNJG3BGXN:2:22103:5162:13677

ACATGGGAAAATGCTGGTTCGTATTGATGTGGTTGGGTGCTACATTTACT

>NDX551268_RUO:41:HNJG3BGXN:1:21111:8593:8564

AAACCTTGGGAAAAAAACAAACCAGTCAATGAAGAAGATACTCCTAAAAG

>NDX551268_RUO:41:HNJG3BGXN:2:23103:23411:9016

TTATGTTTCTGACTCTACTGGTAAATTAGTATTCAAGGAAGGTGTTTTAG

>NDX551268_RUO:41:HNJG3BGXN:2:11104:25070:14126

ATCCTGAAGATCGTGCCAAAGCTAAAGATTTGTTTGAGTCAATATTTGGT

>NDX551268_RUO:41:HNJG3BGXN:1:21107:21786:7207

AGTGACATGATAAGTGGTGATGATGATGATGATAATGAGGAGCAAAGTAA

>NDX551268_RUO:41:HNJG3BGXN:2:13111:23069:10693

TTGCAAGTCGCGTTGAATGTTATTCTCCCAAACTACTATCCCGCTTTCAC

>NDX551268_RUO:41:HNJG3BGXN:2:11307:25098:4962

AGATTGGGCTGCTTGTTGTGCTGGATCTGCAGCATGTTTGTTAACTTTGT

>NDX551268_RUO:41:HNJG3BGXN:4:12409:15175:19667

TCATTTTTGGGAATGATTAATCCCATTGATGATAAATATATCTCCATATC

>NDX551268_RUO:41:HNJG3BGXN:1:21105:24016:15454

ATTATTTCATACAACTCCGCAATTCAAACAAGATGAATATAAATAGAGAT

>NDX551268_RUO:41:HNJG3BGXN:1:12101:16932:4655

AGGAGTAGAACAAAAAGTTGAAGAATTAGAGAATCAATTAGCAATAGCTA

>NDX551268_RUO:41:HNJG3BGXN:1:22304:18337:1770

TATCAATAATAAACCATGGTAAACATCTACCGAAATACATAACTTCATGA

>NDX551268_RUO:41:HNJG3BGXN:2:13207:25814:19508

GACTCGTCATCTAGGTAATCATCACGTCTAATTAGGATTTAGAAATATAT

>NDX551268_RUO:41:HNJG3BGXN:3:13602:4461:19376

CTATAAGATGAAAAAGAAGAAGTCAAGAAAAAAAAAATTAGTTTCTAATC

>NDX551268_RUO:41:HNJG3BGXN:3:21410:24762:7989

AACTTGGCGTTGGACTACTCTCTTCTTTTTTCAAAATGACCTCCTTCTTG

>NDX551268_RUO:41:HNJG3BGXN:4:22508:4204:13609

AATGTCGAATTTACTTGACTTGATAAATCATGATGACGATGACAGTTACT

>NDX551268_RUO:41:HNJG3BGXN:4:13505:22284:2658

TGGTTTGAAGAAAGTCTCACCTTCGGAAATGACACACAAGAATCCTGAAT

>NDX551268_RUO:41:HNJG3BGXN:1:12310:24481:9719

GCATTGTCCTTGATAGACTCCGCTCCCAGGTGGATAAGTTAACCGACGAT

>NDX551268_RUO:41:HNJG3BGXN:1:11305:9995:9175

ACCGTAGATAATGACTACATAATAAGTATAAAATCGACACTGTCTTTATT

>NDX551268_RUO:41:HNJG3BGXN:1:12302:17359:6080

CCATTGACTGTGGAATTTTCTAGTTATGTACTAACAAGAT

>NDX551268_RUO:41:HNJG3BGXN:3:11606:17101:6942

CCATTACATATGGCCCTTTTCCACTAGTTTTTCTCATCTTTGTAGCTGGT

>NDX551268_RUO:41:HNJG3BGXN:2:21104:14383:11499

AATGTATACACGGTGTATTCAGAATCAGTACTTCCTAATCCCAATGTAGA

>NDX551268_RUO:41:HNJG3BGXN:1:12112:17561:10311

ACATAACAGTCGTTCCCTCTCATAGCACAATGTCTTCCATCTTTGGTTCT

>NDX551268_RUO:41:HNJG3BGXN:4:22603:6962:10950

AGAAGGAAATGAAAGACAAAAAAAAATGAAAACAACAAACTGTCCTAGGT

>NDX551268_RUO:41:HNJG3BGXN:4:13504:10517:9246

TCCCCTTGGCTTCTCATTTGCCTCTTTTTCGGCTTCGGTAGCATCAACAG

>NDX551268_RUO:41:HNJG3BGXN:3:21605:10866:3360

GGTTGGATATCCCAACTGTTGAATTGGTAGTAAATTTTGATATTCCTGCT

>NDX551268_RUO:41:HNJG3BGXN:3:11606:26004:16553

GCTGGTATCAACACTATTAGAGAAATCTTGTCCAGAGCTCCGTTAGCTAT

>NDX551268_RUO:41:HNJG3BGXN:2:11101:4302:15375

TCAAAGAGGAGGAGGAAATAATGGATATTATAATAATAATAGTTATAGAG

>NDX551268_RUO:41:HNJG3BGXN:2:22209:18862:3769

GTTACTTGCCTGAACTTTAAAAAGGGAAGCTAAAAAGTAAAAATCCCCTA

>NDX551268_RUO:41:HNJG3BGXN:1:22204:20172:6356

CATCCTCTTTGTTTCTCCGCCCCCTAACATTATTACAGTATCACCAACCA

>NDX551268_RUO:41:HNJG3BGXN:2:12312:22873:3957

TCGCCTTGATGCTACCACGCTATTAAATAACATGTGAATGATTGCTATTG

>NDX551268_RUO:41:HNJG3BGXN:4:23508:10404:15842

AATTAAAAGCATCAAATAAACAATTACTATTTTATTCTTTTTGCTCATTT

>NDX551268_RUO:41:HNJG3BGXN:2:22306:3411:11018

CAGTAAAAGAATCATTATCTTTAGATTCATATTCCGATCCTCCAAAGTTT

>NDX551268_RUO:41:HNJG3BGXN:1:13207:17811:16556

CTATTAGACAATCGACTTTCATGGCCCTACTAGTCACACCCATAGACTTA

>NDX551268_RUO:41:HNJG3BGXN:2:12302:9017:18041

CGTGAAGCAATCAATAACACTTGTTCTGAACTATTTCATAGATCATATCA

>NDX551268_RUO:41:HNJG3BGXN:3:13411:21606:3942

CAAATGGTCTATCTGGTAGCCACCATAACAATGAAATACCAACAGCAATC

>NDX551268_RUO:41:HNJG3BGXN:1:12307:2066:17432

GGTTTGGTATAAGAGTTGCTTCCATGATGTGTCAATCTGTTATCTATAGT

>NDX551268_RUO:41:HNJG3BGXN:3:12406:17339:16057

TCCATGAATATAGAATAACGGCATATTCTTTATATGCTGCTGTGTCAGTT

>NDX551268_RUO:41:HNJG3BGXN:3:23608:7322:10455

GGATTAAACCATGTTTCTCCCCATAACTCATTTCCGTTGTTTTTAAATTC

>NDX551268_RUO:41:HNJG3BGXN:1:13203:1816:5899

GTAAAATGAGGTACATTATCAAAATAATCAAATCCTTGAACTATTGAAAT

>NDX551268_RUO:41:HNJG3BGXN:1:11110:11584:17485

ACAAGAACGTGCATTACAAGCTAAAGAATCAATAGGTGTTGAAACTAAAG

>NDX551268_RUO:41:HNJG3BGXN:1:13204:13629:3669

AAATATAGCAGAAACTTCATTAACTGTTGATGGGATTAAATATGTTATTG

>NDX551268_RUO:41:HNJG3BGXN:2:13211:12031:13508

TAGAGGAGATTGTAAGAGAGTAGAATATCAATATCAGATGAGTCTTAGAC

>NDX551268_RUO:41:HNJG3BGXN:3:22501:7413:10714

CATGATGACGATCACATTGATGTTGGAAGATATTAACCAGGAGATAAGAA

>NDX551268_RUO:41:HNJG3BGXN:2:12303:22229:16729

TAAATATCAATTGGAATTCCACCAAGAATTTTTATTTCAAATTAGTTTAC

>NDX551268_RUO:41:HNJG3BGXN:4:11603:23065:14004

GTGATAATGGAGGTCGAAAAGTATCTGATAATTCTGATAGATTAAGTTTA

>NDX551268_RUO:41:HNJG3BGXN:1:13103:4786:10431

AAATAGTAAGTGAGAGGAAAATCTGGTATCTGTGAATTTACCTTTGCGGG

>NDX551268_RUO:41:HNJG3BGXN:1:23107:13186:13850

ACTGAAACCAACGGGCCAGATGGTACTGATACTGTTGTTGAATATGTACC

>NDX551268_RUO:41:HNJG3BGXN:4:12605:19029:2152

ACGTTTATTGCATCGAGCAGTAATGGGTCCTTGGTTGAATTTTCAATCAC

>NDX551268_RUO:41:HNJG3BGXN:3:11507:23683:4633

GGATATAACAAATACTAATGAAGTGGTTAGATCATTCATTGATTCTCATC

>NDX551268_RUO:41:HNJG3BGXN:1:11110:3813:4760

TTGTTAGTTTTGTCCAATACTAAAATGGTAATCACATGTGGAGAAGATGG

>NDX551268_RUO:41:HNJG3BGXN:3:23607:22241:4128

AACACTCTTTCTTTTTTTATTTTTTTTTTGGAAAATGGTCGACTTAAATA

>NDX551268_RUO:41:HNJG3BGXN:1:11202:5390:1960

ACATTTAAAAACCAAAAGAAAAAAAGAGCTCATGACATCAAGAACAATGA

>NDX551268_RUO:41:HNJG3BGXN:1:23101:1243:11911

ATCCAAAGCGTTTGACACCAAGTCTGCAGAACCACTAGATGCGTTGACTT

>NDX551268_RUO:41:HNJG3BGXN:4:21405:17464:19944

AGACGAATGATGCCATTGTATAATACCAAAGTTGAAAAGACAGAAGCTCT

>NDX551268_RUO:41:HNJG3BGXN:1:13302:19905:6853

CACCAGAGTGATTGTCACCAATGAAGGTGCACAAGAACGCGTGTTTTCAC

>NDX551268_RUO:41:HNJG3BGXN:3:11409:13801:15923

TTAGCCATTGCCACATATATATTGACCACAGTTTCTGGATACCCTATGGA

>NDX551268_RUO:41:HNJG3BGXN:4:11508:18382:12688

GAAGAAGATAAAGAAAGAAGTCTTACTTGGCCAGGATTGTCGATGAATAA

>NDX551268_RUO:41:HNJG3BGXN:3:12407:23924:12179

AAGCAGTCACCACCAAAAGGTCGTATTATAGTGTTGACTCGATTTGGTAT

>NDX551268_RUO:41:HNJG3BGXN:2:13311:2518:14200

ACAAACTACTGGCTACAATGCCCAACCATTGCAACAACAAAGAACTGGAG

>NDX551268_RUO:41:HNJG3BGXN:2:21107:26758:6403

TAATAAACCTGCTTTAAATCAAAGTGAAGTAAGTGCTTTACGTTGGGCTG

>NDX551268_RUO:41:HNJG3BGXN:4:23509:1715:12443

ATATTTCCGGATGGTTTGAATATCCACCATAATTGAATTCAGTGCCTGAT

>NDX551268_RUO:41:HNJG3BGXN:4:11608:10479:10059

CTGTATAATTCTTCTAGTTTAGGGTCAGGACGATAATTTCTAATAAAATC

>NDX551268_RUO:41:HNJG3BGXN:2:12101:16493:15306

GTATTAAAGAGGACGAAGAAAAAAAAAATTTTCTCCTCTGGCATCGTTAC

>NDX551268_RUO:41:HNJG3BGXN:1:21107:21858:9113

TTCTTCCTCTCCTGCCTGTTGAACAGGTTCTCCATTGATATTACTGGTAT

>NDX551268_RUO:41:HNJG3BGXN:3:21608:12862:3439

CCTCCATGTCTTTCTATTAGTAACTTGATAGAATTGGCTAGTCTTTGTGA

>NDX551268_RUO:41:HNJG3BGXN:3:12411:10416:20190

GTTTTAAGATATTCTACATGCACACACACCTTTGTACGCATATGCACGAC

>NDX551268_RUO:41:HNJG3BGXN:1:23104:12924:6585

TTGGAATACCCACCCAAATATAACAACGTCGTTATGAAATTCGCAGTACG

>NDX551268_RUO:41:HNJG3BGXN:3:11603:17299:2775

CAACGGACATGAACCTGTGACCAAATACAGATAACTGGATCGGGTCAATG

>NDX551268_RUO:41:HNJG3BGXN:1:23106:22844:7031

GGCTCATTGTCACCAAGTCATCCACAAAGTCAACATCTGCAGTCATATTA

>NDX551268_RUO:41:HNJG3BGXN:4:23506:4881:18072

TAAGGAAACATTTTGCACGTTGCAATTGATGGGAACAAGTGTATGGCCTG

>NDX551268_RUO:41:HNJG3BGXN:3:13412:22238:5060

AATGGAACCGAAAAGGTAATTACAAATCCTGATGAATTCCCAGATAATTC

>NDX551268_RUO:41:HNJG3BGXN:1:12207:1706:19062

ATTCATGAATGCAATTTTTCCAAAACCAAGCTTCTCACGTAATTGAAGTA

>NDX551268_RUO:41:HNJG3BGXN:4:13511:13368:2750

ATAAATGAGTTCTCCAAAGAATTCAATTGATTTGGCGATTGTTCAACAAA

>NDX551268_RUO:41:HNJG3BGXN:4:11508:9356:13077

CCTGATTAGTAGGGTATGTGGTTAGAAAATAATGTTCTATAGATTGAGAT

>NDX551268_RUO:41:HNJG3BGXN:1:11303:22291:17111

ATTGAGTCAGCCCCAGTTGGATATACACCACATTCGATTCATAGTTATTT

>NDX551268_RUO:41:HNJG3BGXN:1:12201:12682:7302

CTGTTCTCTACTAGATGTCAATAATTTTTCATTTGATGAATCTTTCACTT

>NDX551268_RUO:41:HNJG3BGXN:1:22111:9860:13655

TCAACGTTTTGAGATACAAGTACCTTGTATTACGTTTAAATCCGATATTC

>NDX551268_RUO:41:HNJG3BGXN:4:23608:15085:5084

TCCATCTTATCAATACATCATTACCATTACTAAGTTATGTTCAATAGACC

>NDX551268_RUO:41:HNJG3BGXN:2:23106:4112:8539

GTGTTAACATGCAGCTACTCAGAAAATGAAAAGTTGGTTGCTTCTGGTGG

>NDX551268_RUO:41:HNJG3BGXN:4:23403:16142:9452

ATGGGCAATTGGTTGTATCCTTGGTGAGATTTTAAGTTTAAGACCAATGT

>NDX551268_RUO:41:HNJG3BGXN:2:13105:20224:5102

TATTGATTTCATAACTACCAATGAAACTTCACTCAACCACTATATTGAAA

>NDX551268_RUO:41:HNJG3BGXN:3:23409:24389:2704

AAGATTTGAAACAAGTTAGAGATATTTTGATTCCATTAGGTGAATATTTC

>NDX551268_RUO:41:HNJG3BGXN:1:11112:1300:11211

ACCAGGAACAATTTCCCAATTCGAAAACACTAACGACATTATTGCTTGAC

>NDX551268_RUO:41:HNJG3BGXN:2:12102:6371:9106

ATCTCATCTAAATATTCACAAGAAACTGTCAACAAGGCTCAGGCTAAATT

>NDX551268_RUO:41:HNJG3BGXN:4:11402:7167:10001

CCATTCAACTTGAGGACAACATTCGTTACTAGTATTCAAGTTATTGATTC

>NDX551268_RUO:41:HNJG3BGXN:3:12504:2219:18221

ATCTAGCACCAAAAAACCAGTAGTTAGTGCACCACCGGTTCCTTCTATGC

>NDX551268_RUO:41:HNJG3BGXN:4:22504:14772:3349

TTCTTTCACTTGCTTTCTTTTTTTTTTTCTGCTGCAAACAGATTTTTCCG

>NDX551268_RUO:41:HNJG3BGXN:2:23210:18488:19002

TTTTAGGATCACGGTTGAATTTTTCAGTTAACAATTGACGATCCTCAATC

>NDX551268_RUO:41:HNJG3BGXN:4:22611:14139:13290

TTAGTTTATACTTATTTGTTTTGATGAATTCATCAAGTTCCACTTTAATT

>NDX551268_RUO:41:HNJG3BGXN:3:11507:16641:7184

TACTTACCCAGTATTTGTGAGCAAGCTTGCTACACCAGAAATCATGAAGA

>NDX551268_RUO:41:HNJG3BGXN:3:22503:21504:13038

GAAAATGTAGGGCAATTTCCACTGGTATCACACAAACCATTATTAAGAAT

>NDX551268_RUO:41:HNJG3BGXN:1:23106:2903:14929

TCTTGATTCATCAGATGGTAAGAATGAAGCCACTAGAAGAATCTTAATCT

>NDX551268_RUO:41:HNJG3BGXN:4:23504:5749:4258

CTTTTAATTGTTTTCAAATATTACAGAATAGTGGGCAAGTTGAATTTGGA

>NDX551268_RUO:41:HNJG3BGXN:3:13409:12711:1349

GTTGTGATAAAGATTCTCTTACTCTATTGCGAATTGGATCCAGTTTTTAG

>NDX551268_RUO:41:HNJG3BGXN:3:13607:1414:13364

TGGTAAAACATCACTACTTTATGTATTCACATTAGGAGAATTTCCTTCAG

>NDX551268_RUO:41:HNJG3BGXN:1:22210:19058:8417

TCCAGTGTTACATTAGCTGTTGGAAATGATGAAACCAATGAGCAAGAGCT

>NDX551268_RUO:41:HNJG3BGXN:2:12206:3154:7738

AAATAGTACATAGCATATATCTTCATTTCTATACATTCATAGCTGGTTGA

>NDX551268_RUO:41:HNJG3BGXN:3:11406:10024:9850

CTATACTCACAGGGAGGAAAACTTGCTGTATATAAAATTCCCGTTCGATA

>NDX551268_RUO:41:HNJG3BGXN:3:23408:15805:5232

AACTGATGAATGTGAATTCCATGAATTATTTTTGCGTTCATCTTTTAGAT

>NDX551268_RUO:41:HNJG3BGXN:3:12510:18097:5682

TTTCTCGTGAATTCTTCTTCTTGTTATCCCATGATATGATTAACCCATTC

>NDX551268_RUO:41:HNJG3BGXN:3:13606:20601:12124

GATTGGAATTTAATTCAGCGGGGTTGGAGTTCAGAGGAGAAGTATTGATA

>NDX551268_RUO:41:HNJG3BGXN:3:11410:3819:11815

CATTGTTAGAACCACTGGATTCATCACCGTTGTCAGAACCACCATTGTTA

>NDX551268_RUO:41:HNJG3BGXN:4:12607:19386:10291

GGAATTGAATCAACCTGAGTGGAATATTTTATCCTCATTCTCTTTGTTCA

>NDX551268_RUO:41:HNJG3BGXN:4:13612:19177:5320

ATTTTAATTATTTTCTGTGTCGTGCAATATGGGAGAATTTTATTGTGCAC

>NDX551268_RUO:41:HNJG3BGXN:4:23604:4202:14579

GGATCGTTTGCTGAATCAATTGACATATTTATGGTTGGATTGTAATGATA

>NDX551268_RUO:41:HNJG3BGXN:3:13611:8209:1318

TGATAATGCACATGTGATTAATGTATACAAGAAGTATAAGAAATCACAAG

>NDX551268_RUO:41:HNJG3BGXN:3:11402:21144:2205

ATCTTCAGATGGATCAACTACCAGCTCAGGTATCAACACTCCAATTGTCG

>NDX551268_RUO:41:HNJG3BGXN:3:23601:19484:7159

GAATATTGTATGGGTGGAGAGTTTTTCCGTGCCTTGCAAACTAGAGAAAC

>NDX551268_RUO:41:HNJG3BGXN:3:12409:7604:4228

CAAGAGCCATGCTTTTTTTAAGGATACCCAGTGGGCATTATTAAG

>NDX551268_RUO:41:HNJG3BGXN:1:22103:19679:6591

GTAGTAGGGCAAACGGTGGTGTATTGAACAGTAACTTCACTGGTATAGTA

>NDX551268_RUO:41:HNJG3BGXN:4:13404:5905:14201

ATTGAAACTTTGGCATCCTCAACTTTACCAATATAAACACAATAGATACC

>NDX551268_RUO:41:HNJG3BGXN:3:21404:5006:14046

ACTTCGATCAATAGATATCAAATTATCCGATAGTAAATATGGATATGTTG

>NDX551268_RUO:41:HNJG3BGXN:2:11308:26309:13103

TTATTATTGAACAGATTATCTTTATATTATGGTGAAGTCTTCTATTTGTT

>NDX551268_RUO:41:HNJG3BGXN:4:23612:13983:19117

TTCTTCCTGCTCGTGATCCATTTGTTATATCATCAGACAATGATAAGAGT

>NDX551268_RUO:41:HNJG3BGXN:2:12110:25443:7343

CATTCTTACTGCATTGACTGCCACAATTGCTACTGTTGCTGGTTATGAAA

>NDX551268_RUO:41:HNJG3BGXN:1:23306:23602:12066

GAATAATAATGAATTAAGTTTTGCAAATAACACTAGTGCTTTTATATTTG

>NDX551268_RUO:41:HNJG3BGXN:2:21308:13155:11846

TTAAGTAAGAAATACAAAGATTGGTCTGTTTGGTACGGCAAAGATATTAC

>NDX551268_RUO:41:HNJG3BGXN:4:23408:24340:11193

GATTGCAGTGCTAACGAGCTTAAACCTTATAACCTTGAAAGTATCAAGGG

>NDX551268_RUO:41:HNJG3BGXN:2:22304:11427:19894

GGATTTAGTCTGGATGATGACTTGGCTAAAGAGATTGCCGAAATCGAAGA

>NDX551268_RUO:41:HNJG3BGXN:1:12308:10659:20248

ATTTAAAGTTATATAATTAAAAACAAAGGAATAAACATCGGGAATATAAC

>NDX551268_RUO:41:HNJG3BGXN:1:22204:12083:11282

AGGTCGGATTATCAGGAGGAAGTGTGCGGATCGTTGTAGGTTACTACGAG

>NDX551268_RUO:41:HNJG3BGXN:4:11407:25006:10148

GATTATTCATTTATCAATATGATGCTATTGGTGATGGTCATTATATGGGT

>NDX551268_RUO:41:HNJG3BGXN:1:11101:8107:11201

TAGAAACAGTTTGCACTAGCTTAATGGAGTTGAAAATTGAAGAACGCTAT

>NDX551268_RUO:41:HNJG3BGXN:4:12602:13281:12081

ATTGAGAATCTTCCAGAAAATTTAAAGGAGTTAAAATGTGAGTCTAATCG

>NDX551268_RUO:41:HNJG3BGXN:2:13308:5964:10584

GTAAAACATATCTCTCGATAACTTGATATAAAAGTTCATTTTTTTCATCA

>NDX551268_RUO:41:HNJG3BGXN:1:13307:20619:14137

TATACGATAAAATAGTAGTAGTGGTGGTAGTAAACTTGATGTTAATGAAT

>NDX551268_RUO:41:HNJG3BGXN:3:22602:7173:11128

TTACAGAAGTCGAGAAAAGTGATCCAATAATAAAATAACGTGCTACGTTA

>NDX551268_RUO:41:HNJG3BGXN:1:21104:15569:4343

TAAGCTCTCTTAGAGGAAATTTATTCCGCTCCACACAAAAAATTATAGAC

>NDX551268_RUO:41:HNJG3BGXN:2:11201:14606:6162

GTATAATAATTCAGCTCTTTCTTCTAAATCACTATGAATGGCATTAGTTT

>NDX551268_RUO:41:HNJG3BGXN:2:23102:19677:2228

GTATTCAATTGATCCTGTTCCGGGGTATGGGTTTTTCCATGGTTATGTAG

>NDX551268_RUO:41:HNJG3BGXN:2:11304:17093:12460

TCAATTAAACTAGATTTTTCTTTCCATAATGTAACACACTTATTCATGAT

>NDX551268_RUO:41:HNJG3BGXN:1:13204:4107:13871

TACGAATTTCTTCTTCAGTCTTGGTTCTATCTGCTGGAACAGCTCTTCTA

>NDX551268_RUO:41:HNJG3BGXN:3:12606:14512:12616

GGTATATCTTCTTCAGGATTTAATTCTTTATCATCAGATACAAATAAATT

>NDX551268_RUO:41:HNJG3BGXN:1:23105:16743:14148

TAATGATGATGTTATAAATGTTAATGATTTAAGAAAAGCTGGGAAAGTGA

>NDX551268_RUO:41:HNJG3BGXN:4:12406:9848:5472

CCTATACCTATTCAATATCTGGATCATTTTAGACTATTACGAGTTAAAGA

>NDX551268_RUO:41:HNJG3BGXN:1:12309:8517:10786

TGATTACAGAGCAAAAACAAGAGATTGAAGAATTGCGTGCCCGAGAATCT

>NDX551268_RUO:41:HNJG3BGXN:3:22506:18332:9583

TTTCTTTTTTAATATTCTTTTCTGTTCTCCCTCTATCCCCTCTAACTAAT

>NDX551268_RUO:41:HNJG3BGXN:3:12512:15151:3896

TGGGGTTGACGACGATGATCTATAGTCATTACATGTATGTATTTATATAT

>NDX551268_RUO:41:HNJG3BGXN:2:21110:22318:11341

CAACTGCTTTGCTTAGTTTCCCAACTGCGTAATCTCGGTAGGAACTAGAT

>NDX551268_RUO:41:HNJG3BGXN:3:11609:3656:3080

ACAACCAATAGATATCACATTCAACCATAGAGCTTCTGTGTTTAATAACT

>NDX551268_RUO:41:HNJG3BGXN:3:21407:5021:9179

TAGTTGTTCATGTATCTGCCGCTATTCTCCACCATTTCATATATATATTA

>NDX551268_RUO:41:HNJG3BGXN:3:11406:4339:10360

TTCAACCACCAAAAGGTCAAACTTGTTCTTCATTCTTGGATCCTTATATC

>NDX551268_RUO:41:HNJG3BGXN:2:22210:9708:14270

ACTTTATATGTATATTATAGAATAATATAGGCAATATATCATCATATCAA

>NDX551268_RUO:41:HNJG3BGXN:4:13407:13049:17964

AAATTAGCCCAATCGCCAACGTGTTCTGAATCATAACCATACTCCGAAGC

>NDX551268_RUO:41:HNJG3BGXN:3:12610:17232:18054

GATCATCATGACCGTTGACAGCATTAGCTGTCAAATTTTAGTATAGGTAT

>NDX551268_RUO:41:HNJG3BGXN:3:22510:17784:13323

GACCCGTAGTAGCCCATAATCTCAATTGCCTGTCGTACTTTGTTTCTTTA

>NDX551268_RUO:41:HNJG3BGXN:1:21305:5585:7383

ACCTTAAGCATAGGGGCCAAAAAGTACAATATAGAGAGACTAAACCCACA

>NDX551268_RUO:41:HNJG3BGXN:3:12606:16160:6662

GGATTAATGGGTGGTTCATTTTTGGATAAAGATGATTTCACTCAAGAAGC

>NDX551268_RUO:41:HNJG3BGXN:2:21310:18354:15467

TAGAATGACAACAGTTATGATTGGCGATACATTAACTTCAGATATTAAAT

>NDX551268_RUO:41:HNJG3BGXN:2:11204:14090:10006

GAAGAATCAAGTTCAATACTGCAAAGAAGTCGTAACAATCAACAGTCATC

>NDX551268_RUO:41:HNJG3BGXN:4:23612:10933:4546

ATATTAAGCTTGAAGACAGTATTACAAAACAAGTTGCATATGTAACAACA

>NDX551268_RUO:41:HNJG3BGXN:1:23207:17739:14506

GTGTACATGCCATTTATTCCTGTGTTGCAACAAAACAACAATGGAAGTAA

>NDX551268_RUO:41:HNJG3BGXN:1:12110:3002:17063

TCTGCCTTAATGGAGAATTTGTCACCAAGTCCTATGGTCAACACAATAAG

>NDX551268_RUO:41:HNJG3BGXN:3:12410:21250:8524

TTGCAACATATTGGAATGTAACGGCAAAACCTATAAATAACATCACAATT

>NDX551268_RUO:41:HNJG3BGXN:2:22303:22977:7866

ATCCAATATTGATTCTGAATCAGGAGTAATTGTTGGACTTAACATTAAAC

>NDX551268_RUO:41:HNJG3BGXN:2:13307:25197:19454

GTACAAAAGATATATCTCTGTAAAATCGTTGTTTTGACAGATAGTATTAT

>NDX551268_RUO:41:HNJG3BGXN:1:21309:23441:15204

TAGTATTATTCCTTGAGTTAATGACAGTGCTAGCTACTAAGGAAAAGAAA

>NDX551268_RUO:41:HNJG3BGXN:3:22409:19663:13741

ATACTATTTCACTATTGGTGAAGGAGAACCAGGTATTTTATTGGCTACTC

>NDX551268_RUO:41:HNJG3BGXN:1:13302:14108:10875

ATATTGCAATTTTCCCCCATGTATACAACTAGAGTTTACTAA

>NDX551268_RUO:41:HNJG3BGXN:2:12105:20135:12745

TCTTTTTTCAAGAACTTGCATGAATCTAATATGACTTACAGAAATGAAAC

>NDX551268_RUO:41:HNJG3BGXN:4:22405:15650:9514

GGCAATATTGTTGCTAGATATAGTACTTTTACATCCCCCGAAGCTATTAG

>NDX551268_RUO:41:HNJG3BGXN:1:12107:10335:11634

TCCAATTCCAACCGACAACCTGAAGCTCAATCTGCCAACAGATAGAATCT

>NDX551268_RUO:41:HNJG3BGXN:4:21511:24539:19367

TGAGATATGAGTATATTACTAGACGAAGCAACAACTAATCATGATAGACA

>NDX551268_RUO:41:HNJG3BGXN:2:21105:20007:13029

CTTATTAATTGCAAAAGAAATATCCATGGAACAATATTTAG

>NDX551268_RUO:41:HNJG3BGXN:1:12204:14343:5260

GAAGTGGTTCTCATGTCACGTCCTTCTAACTGACTTAATCTACCTTCAAC

>NDX551268_RUO:41:HNJG3BGXN:2:12208:23334:4901

GAGGTTGTTTATTATTTGACTTCCCAAAAATGGCAAAACGCAAAGTTAAT

>NDX551268_RUO:41:HNJG3BGXN:1:23105:3142:5998

TTTTTTAAGTGCTATCACAGGGGGATTTTTTCCAAGGTGTGTAAA

>NDX551268_RUO:41:HNJG3BGXN:2:23303:24019:8342

TGACCCAGAAAAAGCCATTAACATCTGACCTTGTTTTCCCTGCTTTGGCA

>NDX551268_RUO:41:HNJG3BGXN:4:23504:3617:12770

CAGCAAGAGGGCTCCAAGTTTGACAAGAGAGCATTGTTTACAGCAAAAAG

>NDX551268_RUO:41:HNJG3BGXN:4:22511:8789:16149

CAGATCCAAATAGATGCCGAGCTACCGTATGTCACGAATCGATCAATAGC

>NDX551268_RUO:41:HNJG3BGXN:3:13403:25473:19329

TAAGAAGATACGCAAGAGTAATGCGCCGTTTGGAGGAATTCAGTTAGTCG

>NDX551268_RUO:41:HNJG3BGXN:2:13109:1870:11315

CCATTACGAGTTCGAGTCTCCGAATATTTGAAAGCAATTTCAGATCTCTC

>NDX551268_RUO:41:HNJG3BGXN:2:21302:10732:2149

GAATATCTTAACAATGCTGGTTTTATTACCAAATGCATCATATAAACTTC

>NDX551268_RUO:41:HNJG3BGXN:1:22309:24554:16310

TAATGATACTAACAACGAAGGAGCAAAGAATGCCATACTCAGTGCATTGT

>NDX551268_RUO:41:HNJG3BGXN:1:12207:9636:19096

CAATGGACTTCCAAATTTAGAGAAATCGGCTTCCTTTTAAATCATTGATG

>NDX551268_RUO:41:HNJG3BGXN:4:12402:16085:14614

TAATTATATATCTAATCCAATCGTTGCAGAATTTGAAAAGATGGATACCT

>NDX551268_RUO:41:HNJG3BGXN:4:21404:18138:12711

CCTCTTCAGAAGATGCTGGATCAAAAGACGCCGAAGTTTGTGTTGTTGGA

>NDX551268_RUO:41:HNJG3BGXN:1:11305:26710:7758

TCCTCGTTTCAACAATGACGAGTTGTTTTTCACTTCAAGATTACTTGAAC

>NDX551268_RUO:41:HNJG3BGXN:4:23508:9395:13437

CTCACTCACTCACTCACCAGAAGAAGAAAAGAAAAAAGAAAAAAAAAAAC

>NDX551268_RUO:41:HNJG3BGXN:3:21411:5554:8940

GTAATATCTGAGTCCATATGTTGGTCAAAGCCCTTGAGCGCATTTCCATA

>NDX551268_RUO:41:HNJG3BGXN:1:21103:7654:19788

GCTACTTTGAGTCAAGCCATGGTTCATCCAGTCAAACCAGCTAACGGAGA

>NDX551268_RUO:41:HNJG3BGXN:1:23202:3336:19145

TTATTGATCTGCTTCTTGTATTTAAGAAGTTCTTAGATCTAGAACAACAA

>NDX551268_RUO:41:HNJG3BGXN:1:23312:21035:17079

AAGTGCCCCAGGTCCATTAGTTAAAGTTGTTGGTATCAATGTTCCATTAG

>NDX551268_RUO:41:HNJG3BGXN:3:12603:20797:9607

TAGTAGTGGCAGTAATGCAAACAGGCCACCGATGACATCCGGATCTATAC

>NDX551268_RUO:41:HNJG3BGXN:1:13109:2746:14783

GGATAATTCGAAGTCAAACCAACGTTTATAAACAAGTTAGGTAGATTTGC

>NDX551268_RUO:41:HNJG3BGXN:4:22407:22225:12248

TTTCTAGAGCAGGGTGGGAAACACCTAACGCTACCAAAAGACCATGGTTC

>NDX551268_RUO:41:HNJG3BGXN:2:11202:24083:18595

TGATGAAGGAGTTCCTGCTCATGAAAAGGCTACCAAAGTTTTTGCTGAAT

>NDX551268_RUO:41:HNJG3BGXN:2:21312:16406:13598

ATCTGAAAGTGTTACTAGTGATACATTTACAGAGTCCCGGTCACAAGGTG

>NDX551268_RUO:41:HNJG3BGXN:4:13608:2393:9008

AAACAAGAGTTGTTGCAAAAATTAGACTAAAATTACTGGAGAAATCAAGC

>NDX551268_RUO:41:HNJG3BGXN:2:13107:3960:7211

TAACAATAAAGATCGAATTAAAAATGTGCTACTTGATAAAGTAAAGACAA

>NDX551268_RUO:41:HNJG3BGXN:3:12603:21197:7229

GATGTAATATTGGTGGATACATATAAACCAATGGAATGCATGCAAAACAT

>NDX551268_RUO:41:HNJG3BGXN:2:13307:6959:7060

AAATATAGCACGTCTCTCACAGTGAAACATTGTTTCACCAATTTGATAGG

>NDX551268_RUO:41:HNJG3BGXN:1:11111:13992:8853

TCATCATCATCATCAGAAGAAGAAACCATAATCAAGCCAGTGTTCATATC

>NDX551268_RUO:41:HNJG3BGXN:2:22110:20283:17994

GGTTAGTATCCTATTTCAGTTGAAAATATCAACAATTGTAATGGTATAGT

>NDX551268_RUO:41:HNJG3BGXN:3:23612:25377:1983

ACCGAGAACTATCAACCATATGAGTCAATCCCTATCAACAAC

>NDX551268_RUO:41:HNJG3BGXN:3:13401:14328:1149

CGATAAGTATACTCGTGTGTATGTATGTGTGTCGTGTAAAATCACTGAGG

>NDX551268_RUO:41:HNJG3BGXN:4:22604:25931:15079

CAAGACCAAGGACATAACTGGTGTTGGTTTGGAAACAATGGCAACAATAA

>NDX551268_RUO:41:HNJG3BGXN:3:11502:8359:19393

TTTGGTAATTTGTTTAATATCGTGGCAGTTATTAAATCTCAATATAAATT

>NDX551268_RUO:41:HNJG3BGXN:2:11202:16502:9134

TCACTGAATTGTGTAACCAAATTTTGAAAGAAAACATTGAAGAAAAGGAA

>NDX551268_RUO:41:HNJG3BGXN:2:13310:10916:15225

TAACAATACTCAAACCATTTCACCAGTACCAATTCTTGCCAAGGCTTCCA

>NDX551268_RUO:41:HNJG3BGXN:1:11211:25587:19365

GATCTTTCATGAGTAGGTTTGAAACTTCCCTTTCTAGGTAAACTGGATGT

>NDX551268_RUO:41:HNJG3BGXN:1:13103:14453:19248

GCTAAAGTGTCATGGTTGCGATAAAGAGAAAGTAAAACTAGAAAAAAAAA

>NDX551268_RUO:41:HNJG3BGXN:3:23505:9219:11873

TCCTAATACTAATACTCCTGTTCTTGACATCGTTGTTGTTACTATTGAAA

>NDX551268_RUO:41:HNJG3BGXN:2:11211:16012:19411

TTATATATAGTCATTAAGAGCTGTTCCCAACGAACAAAAAGAATCAACTG

>NDX551268_RUO:41:HNJG3BGXN:4:22502:10229:9066

CTCTTTGTGGTCTTTGAATCCCCCCCACACTGCCCCCAATTCACATACAC

>NDX551268_RUO:41:HNJG3BGXN:2:23105:13531:10798

ACTAAAACTCAACCATTCCCAAATCCACCACCACCAGTTTATCATAGAGC

>NDX551268_RUO:41:HNJG3BGXN:2:13204:6697:5662

CAACCACAAAACATCTATTGATAAATATGTCTAATACACCGGCTCGTTCA

>NDX551268_RUO:41:HNJG3BGXN:1:22210:22882:13691

GTGACTTATCCATCAGTAAGATATAATTGTTGATGTTCGATGTCCATGTC

>NDX551268_RUO:41:HNJG3BGXN:4:11606:12173:9891

GTCCATGTCTAACTTGTTTCAATCTTGAAACATCATTTCCAAATTCATAA

>NDX551268_RUO:41:HNJG3BGXN:1:23309:15458:13356

TGTTTATATTACACACTTCCAAAAGCAGACTTGGAGGAGAGTTTCTTGCC

>NDX551268_RUO:41:HNJG3BGXN:3:11510:12900:4592

ATATTGTACCTATTTAAGGTGGCTCGTTACATAATATAATTGTCATCTAA

>NDX551268_RUO:41:HNJG3BGXN:4:13606:10533:11280

GTATCTTCTGTAACCACTTCTGCGACAATACCCGCTTCTCTAGCTTGTAA

>NDX551268_RUO:41:HNJG3BGXN:1:13110:20597:7185

ACAAAATAGTTCAGGATTTATGATTAAACTGGCATTTAACAGACAAAAGC

>NDX551268_RUO:41:HNJG3BGXN:2:22109:10764:17100

CATTGAACTTTGGCATTATTAAGTGTAATTTCGGCAATAGAAACTCTTTT

>NDX551268_RUO:41:HNJG3BGXN:3:21604:24749:19625

GTTTAGGGGTAACAATTTTTGCAAATTCTACTGCACCACTCATCTTTATT

>NDX551268_RUO:41:HNJG3BGXN:3:23402:11572:13033

TCCTTCATTTACCACGAATTCGGGATTCTCGACTCCTCCTAGAGCAGGAG

>NDX551268_RUO:41:HNJG3BGXN:2:11211:7566:10585

TTGCTGTTTGGGGGTTTGATAAGTTGATACGATTGGTACGAGTAACCAGC

>NDX551268_RUO:41:HNJG3BGXN:1:21305:16270:9439

GTTCTTTCTTGTAAACCACAACAAAACAATAATCATTAATAATACAAAAA

>NDX551268_RUO:41:HNJG3BGXN:4:23407:1669:11722

CGTCTACACAAGTGATTGAAAAAACACAGGTAATTGCTAAACCAAGTGGA

>NDX551268_RUO:41:HNJG3BGXN:1:13103:13855:14463

GTTGAAGTCAAATAAATTAAATGATCCTGACCATGCTATACTAGAAGAAT

>NDX551268_RUO:41:HNJG3BGXN:1:12307:3037:2193

ACACTTGGATTAACTTCTATTAAAACACCTCTTGCAGTTTGAATATATAC

>NDX551268_RUO:41:HNJG3BGXN:4:21505:4516:15702

TTGTTGTCTCTCTAATTCAAAGGTCACAACATTTTAATATTTCTATGGGC

>NDX551268_RUO:41:HNJG3BGXN:4:21604:7458:15736

CATAAGAATTGTCAATGTTAGGTTCTGGTAATTCAGATTCACCAATTGGT

>NDX551268_RUO:41:HNJG3BGXN:1:21208:2554:18737

AAAACCATGCTTCTTGTTATAATCATCAGTTTCTCTTTCCCATTGATTTC

>NDX551268_RUO:41:HNJG3BGXN:2:23312:10332:14954

TACTGGATATAAATGATAGTTTTGCTAGACCAAATCTGAAAGCTTTAAGA

>NDX551268_RUO:41:HNJG3BGXN:4:11406:8836:7941

CTCTTTGGGGGGTGGCATAAAATTGTTTTAAAAATTTTGGGATGAAACAA

>NDX551268_RUO:41:HNJG3BGXN:3:12403:5964:18730

CAATTCTTCTGTTTGTTTAAATATTTTCACTTGTAATTCTTCATCAGTAT

>NDX551268_RUO:41:HNJG3BGXN:3:22610:20851:10863

ACATTACATGGTGTTTGATCTTTTCTTTCATATGCCATTAATTGTCGTTG

>NDX551268_RUO:41:HNJG3BGXN:2:11310:7811:8663

TTTGGTCATTTTAATGTGATGGTATTAATGTTGATTGGATTCTCCCTCTC

>NDX551268_RUO:41:HNJG3BGXN:4:22607:18395:12269

TGTGAACACAAACCCAAAACGTAGCCACAACTATAATAGTAGTTATTTAG

>NDX551268_RUO:41:HNJG3BGXN:1:13206:2027:16777

CCTGAAAAATTACTAGAAAGTTCCTGTGAAGATCTGTTGATTAAGCATTT

>NDX551268_RUO:41:HNJG3BGXN:2:23108:22324:10389

TAATTAGACCGGCCCATGACAATAGGTTTTGGTCTATTTTTGCCAACACT

>NDX551268_RUO:41:HNJG3BGXN:2:23105:12439:6334

TATAATATTCGAACAATGGAGTTTTTTGGCTTCTCTGTAATTTGATGATA

>NDX551268_RUO:41:HNJG3BGXN:1:23110:18884:10794

GTTCTGGTATTATCGCTAGAAGAAGAAAGAGAGGAGAAAGAAATGAATCG

>NDX551268_RUO:41:HNJG3BGXN:2:22312:19345:8481

AGACAATTTGGAAGAGGTAGACATGGTGGCCAAGCAGGTGCTGAAATTGC

>NDX551268_RUO:41:HNJG3BGXN:4:23603:19011:11714

TGATGGTCTCACATTAGGGTTTGACTTTCTCAAATGATAGTATTCCATGG

>NDX551268_RUO:41:HNJG3BGXN:1:23312:19935:1970

AGAAAGTAATCATTGATAGATTGATCATCAAGCAACATAAAGGTATCAAA

>NDX551268_RUO:41:HNJG3BGXN:1:22308:21853:7559

TTATCAGGATCTTCAAATTTTTCAATTAAAATCGATTCAAATTCAGCAAC

>NDX551268_RUO:41:HNJG3BGXN:4:21409:5582:4269

CTGGAATGATCATCGGAAAGCACCAAATATGACTCTTCGGA

>NDX551268_RUO:41:HNJG3BGXN:2:23103:12330:12587

TCTTCTTCCTCTTCTTCCTCTTCATCATCCTCATCCTCCTCATACACGTC

>NDX551268_RUO:41:HNJG3BGXN:1:11207:12812:9789

GGCTGTTAAAGGATTAGGAGAACTTGACCAAAAATACGACGGTATGTTAT

>NDX551268_RUO:41:HNJG3BGXN:4:22406:14224:17342

TTAATTAAATAAAACGACACAATCAACAAACATATTATTCTAATATATAA

>NDX551268_RUO:41:HNJG3BGXN:4:23502:5112:2454

GATGAAAGCCCCACCAGAAAAGCCACAAAATATGGGATCTTTCCCACCAC

>NDX551268_RUO:41:HNJG3BGXN:4:23611:19464:6891

TACACATGATTCTGCAGCAAAATTGATAATATCAGTAGTATCTCTTGTGA

>NDX551268_RUO:41:HNJG3BGXN:2:12211:1491:6614

GTTTTTGTTTATTGTACCATAGAGATTTTCCCTTCCCAACTATATAAACC

>NDX551268_RUO:41:HNJG3BGXN:4:12501:19360:3811

GTTGAATTAGAAATTCAAGAAAAACAAAGAATTGTTGCTCAGAATGATAA

>NDX551268_RUO:41:HNJG3BGXN:1:13201:1902:19219

GTACATATACTGATCAAAGATTGGTGTATTTAGGTAGCAGAGTTGGTACC

>NDX551268_RUO:41:HNJG3BGXN:3:22512:18944:2727

ACCTTGAAGTTTCGTAATTAGGTGTACTATCATCATGTTGATATTCAGAA

>NDX551268_RUO:41:HNJG3BGXN:1:22304:26784:16333

ATACCAAACTGATCATAGATAGTTTTCCATTTGCCGTAGTTGGTTCAACC

>NDX551268_RUO:41:HNJG3BGXN:3:12603:19764:7019

TCTTTGTACATATAACCATAATCGATCATGAACTTCAAAATGTCGAGGAA

>NDX551268_RUO:41:HNJG3BGXN:4:13601:1653:4589

GTAGAAAGTTTGATGATTGGAAATAAATAATTTTTTAAAAAAATAACAAC

>NDX551268_RUO:41:HNJG3BGXN:1:11207:12568:11841

GCAATTTATTTTACCGGTTCCAAAACCACTGCAAGACGGCTATGAATTGC

>NDX551268_RUO:41:HNJG3BGXN:2:11111:14181:16273

CCTTAAAGGAGTTCATGTTACTCTTGAAGAGTTGTCTAAAAA

>NDX551268_RUO:41:HNJG3BGXN:1:23208:6522:11053

GATAACTTCATTAGTACTATGATTCTCCGTATCATGCATATCGGTTTTAT

>NDX551268_RUO:41:HNJG3BGXN:2:13106:7200:18098

AATTTTATTGTTTCTCACACAAAATACTACAATTCGAGATGGATAGAAAT

>NDX551268_RUO:41:HNJG3BGXN:4:12607:10708:5165

TCTTTACATTCGGCAATTTTTTCATTCGTTGAACTACGCTTTTCGTCGAT

>NDX551268_RUO:41:HNJG3BGXN:3:22401:23597:16585

GTATTCTCTTCATCAATTTGACGTTGTAAATCAGCAATTAAGGACGCTTT

>NDX551268_RUO:41:HNJG3BGXN:2:12106:16135:2943

TGTTGGTACTATCCAATGCACATGTTCAGCATAATCACCACACCAACCAA

>NDX551268_RUO:41:HNJG3BGXN:4:11411:9667:3843

ATATACCATAAAGAAGTTTCTTCCATAATGGAAAATTATATGGA

>NDX551268_RUO:41:HNJG3BGXN:3:23511:25593:10423

GTATTAAAGTTAAAGATGTGCTTGTCAGTAGATAATGGAGTACCCAAGTG

>NDX551268_RUO:41:HNJG3BGXN:4:22602:7670:14269

GGGAGAACCGTATGAGTATTAACGGCAAGTTCGTCTACCCCTGTTTGAAT

>NDX551268_RUO:41:HNJG3BGXN:1:13312:3569:10609

AATCCATAGTAAATGAAAAATGGATATAATCCATGATATGC

>NDX551268_RUO:41:HNJG3BGXN:3:23510:16971:8121

CTCCAAATAGAATATGTTACAAAAATTACTATGAAATGTTCCCCCCAAAC

>NDX551268_RUO:41:HNJG3BGXN:1:11202:13708:16344

TCTGTATTGAAATCACAAAGTACTAGCACTTCCAATAATACCAATTCTTC

>NDX551268_RUO:41:HNJG3BGXN:3:13502:7642:9989

AATGAAACCAGATTACACCAAGTGTATTTTGAGAAGAGCCACTTCTTACG

>NDX551268_RUO:41:HNJG3BGXN:1:11210:5499:2444

AGGTGCTGTTCTGTTCTGTGCTGTGCTGTTATGTTTCCCTGTTTATGGTG

>NDX551268_RUO:41:HNJG3BGXN:2:21212:11547:18201

CACTACTACTGCTACGACTACTACTACTCCCACACGCGGGAATACGATAA

>NDX551268_RUO:41:HNJG3BGXN:1:21101:10525:1203

CATCGATAATAATATTGGCACACACAAAATCCCTGCTATGTTATCAACCT

>NDX551268_RUO:41:HNJG3BGXN:2:23103:2240:17436

CATGGGTCCGCAAACTAAATTTGTCCAATGTCCAGAAGGAGAATTACAAA

>NDX551268_RUO:41:HNJG3BGXN:1:23302:2854:13380

GAGCATTGGAAGATGAGTTTTCTCCTATTGTCATTATGGCTACAAATAGA

>NDX551268_RUO:41:HNJG3BGXN:4:22512:1422:19323

TGATTATCCAAACACGTCTCTTGTGGAAAAAGACAACAAATTCAAACAAT

>NDX551268_RUO:41:HNJG3BGXN:2:22212:10269:14531

CTATAAATGAGAGTGATATTGAAATACATAAACGAACAATAACAACAACA

>NDX551268_RUO:41:HNJG3BGXN:1:13310:8407:4572

ACCGAAAACATCTACAACAGTTTCTATACTGCCAACTCGACGTTTGTGAC

>NDX551268_RUO:41:HNJG3BGXN:4:23603:18751:5792

GTGTTGCTGTACATGCAGGTATAGTTCCACAATTATCATTAAAGAATCAA

>NDX551268_RUO:41:HNJG3BGXN:3:23611:1974:3655

TACTAATAGCCGTCAATTTAAAAGACCAAGATTGGAAAAAGTTTTGTTGA

>NDX551268_RUO:41:HNJG3BGXN:4:13409:11713:2857

TTGGTCAAATGTCCAATTAAGATTGTAGGATTGAGTGAATTACCAGAAGT

>NDX551268_RUO:41:HNJG3BGXN:2:13301:18312:17096

TTGAAGGTAAAAATTCATGATATTGTAGGTTCCTACATGACAACTATAAC

>NDX551268_RUO:41:HNJG3BGXN:4:11607:18474:19402

TAACTGAACTACTTAATATGATCTTGCAGGTCAAAGGTGAAATTAAGATG

>NDX551268_RUO:41:HNJG3BGXN:3:11512:20056:15580

TTATTACCCTTGTTTGATTCTTGTAATATTAGTTCAGGATACCTCACGGG

>NDX551268_RUO:41:HNJG3BGXN:1:13107:21293:12641

ATTCAAATATGATTTAGGTTTGAAGAACTTGCTGAGTAGTCCCTTTGGGC

>NDX551268_RUO:41:HNJG3BGXN:4:23407:11851:13321

TGATGCTTTGGTTCGTTGAAATGTTTGACAAATACTTTTTTCCCTTTATA

>NDX551268_RUO:41:HNJG3BGXN:2:23104:11481:16518

GGCTAAAATTAAACAATTAAAAAAAAAGAACTTCCACATCTGCTTTAAAA

>NDX551268_RUO:41:HNJG3BGXN:2:12111:15927:7765

AATTACCACCAGGTCACAGAAATAGATATTTTGCATGGTTTACCAATGGT

>NDX551268_RUO:41:HNJG3BGXN:2:12211:2864:14040

CTTTAAGAAAACACATTGCTGGGTTTGTTGTTGTTTTTGGTATTTTCACT

>NDX551268_RUO:41:HNJG3BGXN:3:23611:3383:16254

ATCATTCTTGTCACTGTCGCCACCACCAAGTTCATCTTTCCCCCATCCGG

>NDX551268_RUO:41:HNJG3BGXN:2:12204:17293:12964

CCATTTTAGTTTCGGTTATTAGTTTCCCGGCAAATGTATAGTTT

>NDX551268_RUO:41:HNJG3BGXN:1:23303:6970:15346

ATATCATGGACATAATAAAAAACAAGTATCCATTTTGGAAGAGATTACTA

>NDX551268_RUO:41:HNJG3BGXN:3:23604:11037:13323

AATTAACTAAAAACCAATAAACTAGCATCAAATTATTACTAATATATGTA

>NDX551268_RUO:41:HNJG3BGXN:2:11107:1685:12840

ATAGTTTCTTGGACAAACATTCCTGCTGATTCCACTACATCTTCTTTAGG

>NDX551268_RUO:41:HNJG3BGXN:2:12307:15999:10065

TTGTCACATAAACTGTTGTTTGAATATTGGTGTTCTTTAATACCGCAAGT

>NDX551268_RUO:41:HNJG3BGXN:1:22206:11736:10856

AGTTGGAACTTTACCAACAAACGGGTGAAAATGAATAAGCATGCCCGTGT

>NDX551268_RUO:41:HNJG3BGXN:1:12305:20288:16159

TTATTATTACTACTATCAGTATCTACTGTTGAATTCGTAGTAAGGTGAGA

>NDX551268_RUO:41:HNJG3BGXN:2:21311:23337:8814

TCATTATTTGCCAAATATATCCCAGTCAATCCATCATATGATTCAATATC

>NDX551268_RUO:41:HNJG3BGXN:2:23105:13984:7706

CTTCTAGACAATTTGCTACTCGTTCTGTCGCTTTAGTTGTTGCTGCTTTA

>NDX551268_RUO:41:HNJG3BGXN:2:22107:10167:11204

CATATAATGACATCATAATTCAATTTTTCCGCACGTCAAAGGAATCTATA

>NDX551268_RUO:41:HNJG3BGXN:3:11606:11993:6493

ACTCCTTTCCATTCAATCCGATATCTTTGGAGGGGGCGTTTTCAAATATA

>NDX551268_RUO:41:HNJG3BGXN:1:23112:17922:12040

AAGAATTGAGCAATGGTATAAATTCTTGACAGAACCAGATTATATTACTA

>NDX551268_RUO:41:HNJG3BGXN:2:11201:19898:4478

ACAGCATTAAAAGGTGTTTTTGACAATATTCGTGATCTTACCAATAGATC

>NDX551268_RUO:41:HNJG3BGXN:2:23108:22816:7750

GTTTTAGATAAACCAATGGAATCAGTATTTGGATTTATTAGTGTTTTACC

>NDX551268_RUO:41:HNJG3BGXN:4:23512:7753:3336

CATCAAGATACTCAAGTTGATGAAGACGAAGATGATGATGAACGTGATAT

>NDX551268_RUO:41:HNJG3BGXN:2:12110:9321:18324

ATTTATCAAACCAATCCATCAGACACCACCCATTTTGATAAGGCTTTTAG

>NDX551268_RUO:41:HNJG3BGXN:1:13309:9783:17068

TTTTAATAAATCATGCCAGTTTTCGCTCTTGTTGGTTTGTGGTTTAGTAT

>NDX551268_RUO:41:HNJG3BGXN:4:12502:26241:12616

GATGGGTACTATATGAGATGTGGATTTCAATGCATTGTGTGTAAGAGTTC

>NDX551268_RUO:41:HNJG3BGXN:3:11609:8725:7714

GTTCCAGATCAATCTTTACCATCTTCATGTCAAATTGAATGTAAACAACT

>NDX551268_RUO:41:HNJG3BGXN:2:13302:14461:17844

AATAAAGAGTGCGAAATTTTTTATAGTGCGGATTCTACTATTAGGGCTCG

>NDX551268_RUO:41:HNJG3BGXN:3:21605:10540:11289

ATATATTAAAAAGGGGAAGTTGTATATATGGGAACGGGAGCCAAA

>NDX551268_RUO:41:HNJG3BGXN:1:22111:11292:17305

CTAATCATCGCATAACTCCACCAATATTAGTGGTAACCTAGTGGTTTTTT

>NDX551268_RUO:41:HNJG3BGXN:1:11108:22484:1666

TTGGAGGTAACATAATATTCAAAGGTAAAAAGCAACCCATAGCAAATGGT

>NDX551268_RUO:41:HNJG3BGXN:3:22411:19200:18666

GACAATTTCACCATCAGCTGGAATTCTACCACCTGGGTAAACAATAGAAT

>NDX551268_RUO:41:HNJG3BGXN:2:23301:25847:17086

ACCACTACTAGAACAACCACACGAACAAATAATTTCCGACGATATGTGGT

>NDX551268_RUO:41:HNJG3BGXN:1:23304:6401:1715

ACCATACCAAAGGAAATTCCCCATGATGTACGATCAGGCACCTGTTCAGG

>NDX551268_RUO:41:HNJG3BGXN:2:12107:17905:11138

CTGTTTTACGAGATGACTCCGGTAATATTGTTGTATTGGAGATATTGTGA

>NDX551268_RUO:41:HNJG3BGXN:4:11404:21662:15910

GTGATACTCTAGGTATACGAGCTATTTTTGAACAACTTCTTATTAAGGAC

>NDX551268_RUO:41:HNJG3BGXN:3:23408:1677:10752

TGATAGAAAACAGCAAAAAAGATAATGATTGATGAAAAGGCAATACCAGC

>NDX551268_RUO:41:HNJG3BGXN:1:21305:12407:9018

GGGTAAAACTCTAAAACCTGCTTAAATTTCAAGTGTGACAATAAAGTCAG

>NDX551268_RUO:41:HNJG3BGXN:2:12303:14902:5895

TTTCAAAATCATCCCCAGTTAACAGACGGAGATCTGGTTCAGTGATATCA

>NDX551268_RUO:41:HNJG3BGXN:4:21606:25665:15385

CACTACCACCAGCAAGTAACATCACTGTCACCAAGATATTGGTACAGAAT

>NDX551268_RUO:41:HNJG3BGXN:3:22408:24688:17312

GCGCCGGAGAAACAGGGCCGACGCGCATGTGACGCCGCACGGAGA

>NDX551268_RUO:41:HNJG3BGXN:3:11406:18476:18737

CGGAGTTATTGCTACTGAATCTGGTGTCGTCAGTTCATTTGGTGAAATAC

>NDX551268_RUO:41:HNJG3BGXN:2:13311:16194:12277

AACCTTAGAGGAGAACTTAGCAAGAGAATTTGGATTGTTTTTGACGGTGA

>NDX551268_RUO:41:HNJG3BGXN:1:12107:2448:1351

CTCCAGCTCCAGGTACTGAAACTACATCTGCTCCATGAGAAGGTGATGAA

>NDX551268_RUO:41:HNJG3BGXN:2:22309:6277:8640

CACATTGAAAAATCGGCTGTGCCTCGGTTCTTCCTCGGTTTGACCAATGA

>NDX551268_RUO:41:HNJG3BGXN:2:11112:18502:7755

TGGATAAAACCATCAAGACCAACTCTTAATCGAGCCGGTCTGGTTTTCAT

>NDX551268_RUO:41:HNJG3BGXN:3:11404:13358:7852

GGGCCGACGCGCATGTGACGCCGCTCGGAGAAAGAGGGCCGACGGGGGTG

>NDX551268_RUO:41:HNJG3BGXN:3:21409:19867:17313

TAAATGGACAGGATATTTGCCTCCCAACTCAAACAGATTGTTTACCAACA

>NDX551268_RUO:41:HNJG3BGXN:3:11406:25006:17008

CATCAATTGTTAATGACAAATCGTTATTCGATGAAGCAAAATACAAAGAA

>NDX551268_RUO:41:HNJG3BGXN:2:23307:2111:12968

TCCATGTATTGCGTTTAAAAAATGGGGTCCTAAGAAAATTGCAAAATATT

>NDX551268_RUO:41:HNJG3BGXN:4:21607:25223:11134

TGATGATTCTGACTGTGATGATGATGATTATAATCGTGGTAAAGGTAGAA

>NDX551268_RUO:41:HNJG3BGXN:4:11609:12642:6204

GTACTACACCTAGACAAATCAGACAACAAGAGCTTAATCTCAGCA

>NDX551268_RUO:41:HNJG3BGXN:1:22311:10932:4717

TTACTTGGCAGTGTTAGTTCTACAATATGAGAAACTAATTGACACTAGCC

>NDX551268_RUO:41:HNJG3BGXN:3:12411:17310:6426

GTCTAACTAACTACGATATAGTAAAGAAGAACATTTTTTTTTTTGAGCTG

>NDX551268_RUO:41:HNJG3BGXN:3:21504:7100:6739

ACTCTGTTTTTTCTTCTCAACTGGATTAGTCTAGACTGGTCTGATAATAT

>NDX551268_RUO:41:HNJG3BGXN:3:22601:23117:2607

TGATGGAACAGATGAAGAAGGAGGTGATAAATACGAGTCTTCAGTTGGAG

>NDX551268_RUO:41:HNJG3BGXN:4:11512:2712:15691

AAGAAAATGGTGAGTCTATACATGATACTCGAATATGAACAATAAGAAGA

>NDX551268_RUO:41:HNJG3BGXN:2:12310:10922:16097

ACATTACACTCATCCTCACCAACCATCACAAACCCCATCTCCACAATCAC

>NDX551268_RUO:41:HNJG3BGXN:4:21505:8301:15225

TGCTGGATGTATACAAAAAGATAAACTAAATATAGATTGCGGCCGTATCT

>NDX551268_RUO:41:HNJG3BGXN:2:21102:16376:14319

TAACATATATGATCATAATAATAACGTCATTGATTGTTCCCACTATAATG

>NDX551268_RUO:41:HNJG3BGXN:2:22103:24510:11444

GGTAGTAGCAGCTGAAACATCTTCGTATTCAGTGGTAGTAACAGTTGGGT

>NDX551268_RUO:41:HNJG3BGXN:3:12402:1776:15171

GATATAAATTGCTTCTTCAATCATATTTGTATCAATGCTTAAGTTTGCGA

>NDX551268_RUO:41:HNJG3BGXN:2:12209:22083:15907

CTTCCTGACCCCATGGTTGTAGTTTGTTCTACTCGTAAGATGTATTATCT

>NDX551268_RUO:41:HNJG3BGXN:3:11411:23578:6595

GAACTAACTTCTGGGGAAGATCTAGCTAGTCAAATATCTTTTTGGTCAGT

>NDX551268_RUO:41:HNJG3BGXN:4:23609:20204:2932

AAGTATTGACTGAGAAATTGGACCCCAATAGAACTGATTTAGCATTGTAT

>NDX551268_RUO:41:HNJG3BGXN:1:12311:24699:13711

TCTTCAGTGTGCATTGTGAAACAGGCAACCATCCAGGCAATGAATGTAAA

>NDX551268_RUO:41:HNJG3BGXN:4:21404:25714:15168

TCATGGACAATAGACCCTTGTTCCGTCACCAAACTGATGCATGCAGAAAT

>NDX551268_RUO:41:HNJG3BGXN:3:23402:3553:6631

TAAATTACTGCAAAGAACTATGGAAATTGGCACATAGACATATATATATG

>NDX551268_RUO:41:HNJG3BGXN:4:11509:16192:14713

GTTTTAAGATTTCTGTTTTATTTCATTTCAACCGATTATTTGTATAATAA

>NDX551268_RUO:41:HNJG3BGXN:4:23607:2351:14892

CAATTTTTCATCAAGAACATCAGCTACTTGTGAATAACCAGCACCATAAC

>NDX551268_RUO:41:HNJG3BGXN:1:11101:22767:16608

CTTGCTTTGCTTTTGTTTGTCATTTTGCATTGCTACTGTGAGTGTATTGT

>NDX551268_RUO:41:HNJG3BGXN:2:22109:1672:5512

AAATTCGACAAAGGAAAAGATGCGTATGACAATGGCAAGCATTCTTATGA

>NDX551268_RUO:41:HNJG3BGXN:4:22404:19974:5385

TCTTTTAAAAATGGGTATTTCAAGAAAAAGAGAGTTTAATAATAATAGAT

>NDX551268_RUO:41:HNJG3BGXN:1:23111:13170:8998

GATCAAGAGATCATAATATTCATGTTTGGCAATTACCAATTGTTGATGAA

>NDX551268_RUO:41:HNJG3BGXN:3:23609:1835:11976

TAATATTACGTGACGGTTATCAGTTCATACTAAAAATGTATGCTTTAAAT

>NDX551268_RUO:41:HNJG3BGXN:2:22212:16594:1613

TATACAACTAATGTTGAAAGAAAGCAAAAATGTTATGTAACTATACATTT

>NDX551268_RUO:41:HNJG3BGXN:4:13604:14576:9550

TTGGAGGGTTAAAGACAAAACAAGAACAACAACAGAAAAAGCCTCACCAT

>NDX551268_RUO:41:HNJG3BGXN:1:21105:11757:17177

ATGTAACCGGCGCGGTAAGACTGGGACGAGCGTATGACAGCTCCCTGTAC

>NDX551268_RUO:41:HNJG3BGXN:1:21302:25763:15718

GTATATTATAATTAATCTGTGTGTGATGATTTACCAAATAAAAAAAATTA

>NDX551268_RUO:41:HNJG3BGXN:3:21610:24110:11581

GACTAATCATATTACTAAGTCAAAATGCATGGATGATGGT

>NDX551268_RUO:41:HNJG3BGXN:3:11601:4369:7738

GTCTTGGATAAGCAAATCGTGCTGTTGAGAGAAACTCCGCGATGGTATGA

>NDX551268_RUO:41:HNJG3BGXN:4:23501:8323:2370

CAATAATCAACAACCCAACAACAACTTTGACTATACAACTCTATTACCTG

>NDX551268_RUO:41:HNJG3BGXN:1:22104:21601:7319

CTCTAAGGTTGAAATACTGGAAGTCTCTAGTCTGAATTCTGAGAGTCAAG

>NDX551268_RUO:41:HNJG3BGXN:4:21608:21525:1443

TTGTGGTACGGAGATATTGCCACAGGGGATATAATAGCAACTACCTTTAC

>NDX551268_RUO:41:HNJG3BGXN:1:21109:14212:14787

AGATTAGGGATGTGATCTGGAATCGGTGTGAATTATCCCTCTTATTCTCT

>NDX551268_RUO:41:HNJG3BGXN:1:23204:22405:19743

AAATCTTCAGGGATATTTTAATAATTAAAATCAATGTATTAACTCTGATG

>NDX551268_RUO:41:HNJG3BGXN:2:13307:4748:8032

TACGTAATCCACAGTTTTTAAATTCTTTTAGATCAAACATTCATACCCCA

>NDX551268_RUO:41:HNJG3BGXN:3:12609:12852:10332

GAATGAGACCCTACAATAGCTGATTTTACCGGTTGATGAGATTGTCGCTG

>NDX551268_RUO:41:HNJG3BGXN:4:12409:2516:12928

GAATTATATTGTGTACCATTTTCTCTTCACTTCTCTACTCCTCTCTTCTC
