# Supplementary material for: Case report: Successful combination therapy with isavuconazole and amphotericin B in treatment of disseminated Candida tropicalis infection
Source: Front Med (Lausanne). 2024 Jun 24;11:1397539. doi: 10.3389/fmed.2024.1397539 (PMC11228301; doi:10.3389/fmed.2024.1397539)
Supplement: Supplementary file 5 [file Data_Sheet_5.docx]

Supplementary Material

Case Report: Successful combination therapy with isavuconazole and amphotericin B in treatment of disseminated *Candida tropicalis* infection

Qibei Teng^1^, Xueshi Ye^1*^, Bei Wang^2^, Xinyue Zhang^3^, Zhizhi Tao^4^, Xiufeng Yin^1^, Qianqian Yang^1^

*** Correspondence:** Xueshi Ye: Yexueshi2008@zju.edu.cn

# mNGS sequence of C. tropicalis from whole blood

>TPNB500593:305:HWWHGAFX3:1:11207:18416:4558

GTGTTGATGAAGTTGTTTATTCTGATAGTACTTCAAAGTA

>TPNB500593:305:HWWHGAFX3:1:11105:10271:1552

CAATGTCGATGACCCAGTCAATTTCTGTAAATCCATTAAA

>TPNB500593:305:HWWHGAFX3:1:11101:19116:15918

GTTGGTGTCCATTCTATACCAAAAAGGGGCGAATCAGCTC

>TPNB500593:305:HWWHGAFX3:4:21409:15017:17781

GCAGAACCACCAATGAAGATATTGCTCCACCACCCATGCC

>TPNB500593:305:HWWHGAFX3:1:11102:24508:9388

GTATAACGATATGGTATCCAAGTCCTCGCGTATTAACGAT

>TPNB500593:305:HWWHGAFX3:3:11406:23005:8483

TTCCAATACTAATTCCAGCGCCGATATGTTTTGAAATGTC

>TPNB500593:305:HWWHGAFX3:2:21311:1186:12028

ACCATAAACAGGACCAGAATCCACCGGAGGCACCAGTAGC

>TPNB500593:305:HWWHGAFX3:1:11210:5480:5832

GTCTATCCCTAACAAGGACGAATTGTTTTCAAAGATTGTT

>TPNB500593:305:HWWHGAFX3:1:11301:16300:12491

GATACTGATTCACACCACCCATCTCAGGTTCGTTTGGCAC

>TPNB500593:305:HWWHGAFX3:1:11204:15526:17214

GCATTAACCAAGTACGAATCATCATGACCTGTAGATGAAC

>TPNB500593:305:HWWHGAFX3:1:11202:24038:5887

TTGCAAATGGAACCATACACCAAAAACAAGGCTTCCCGTG

>TPNB500593:305:HWWHGAFX3:1:11210:6382:13047

ACAAAGTTCTATGTCACCAATGTATTTTGATCCTCCGCAA

>TPNB500593:305:HWWHGAFX3:1:11203:7515:13560

TGTCATTTTGAGTCCAGATAGAACGTTTGCCGCATTACGT

>TPNB500593:305:HWWHGAFX3:1:11307:12581:4959

ATCTTGAGACAGTCATTGCTTCATGCTCAATTTGATCAAG

>TPNB500593:305:HWWHGAFX3:1:11101:16593:16676

AGATAAATCATCCAGGTAGTTAGCTTTCATTTCCAAGTAG

>TPNB500593:305:HWWHGAFX3:2:21111:6357:17100

CCCGAAATCAGCTATTAATCCAATGAATGAACCTAATAAT

>TPNB500593:305:HWWHGAFX3:3:11505:1585:12786

CTCCACAACGAGGTACTACAGTCAGAGTTGTGGGGAGTAA

>TPNB500593:305:HWWHGAFX3:1:21305:25117:6329

GCGAGTACTTGAAGAGCATCCCTTTCAATTTCTAGTAAGT

>TPNB500593:305:HWWHGAFX3:1:11112:5698:16217

ACGATGTCCATGCAAAACACTGCTATTCAGAATAATGGAC

>TPNB500593:305:HWWHGAFX3:2:11312:18954:17740

TAAGATTAATGGATTCTATCAAGACGGCCTCCGATTAGCC

>TPNB500593:305:HWWHGAFX3:4:11608:12550:13955

GTTGTATTAACGGTTGAAGTGGGTTGTTTTGGTCGTCTTC

>TPNB500593:305:HWWHGAFX3:1:11106:19609:12321

TTCAATTACACAAGTTGTATTTTTTGAAGGCCTCTTGTAA

>TPNB500593:305:HWWHGAFX3:2:21309:11455:17037

AATCTGTACACAATTGATGAAGAAAATGACCCAGAGATTA

>TPNB500593:305:HWWHGAFX3:1:11103:25835:6508

CATTTGGACTATCAATACAGTAGATCGCGGTGTCCTTCAT

>TPNB500593:305:HWWHGAFX3:3:11609:25741:10982

TTACCTAGATACAGGTCTGTCTTGTTATCTGGACCAACAT

>TPNB500593:305:HWWHGAFX3:1:11312:10643:16758

GTGAGATGCGTGAAAAGACCGAAGAGGAACTTGCCAAGGA

>TPNB500593:305:HWWHGAFX3:1:11110:9193:10139

TTTGAAGGATAGAGTTGATCCAAAAGATTGTCCATGGTAC

>TPNB500593:305:HWWHGAFX3:1:21208:17110:2369

GATAATTATCTTCGTGTTAGTTCCTTCTAAGGTACGGATG

>TPNB500593:305:HWWHGAFX3:1:11211:23113:15500

GATACCACCAAAGGAGATGAATAGGTAGATGAGCTTCGTG

>TPNB500593:305:HWWHGAFX3:1:21306:25906:14407

ACAAGGATTGCTGACGGAAACTTCACCTTTACCTTTGGAA

>TPNB500593:305:HWWHGAFX3:1:11108:6034:11415

GTATTTGTCTATAACTTCTGGATCTTCAATCAAGGAATTG

>TPNB500593:305:HWWHGAFX3:2:11211:5619:15529

TTTCTTGCTGAAAAACATGGGTTCTACAAGTCTCGCAACC

>TPNB500593:305:HWWHGAFX3:3:21607:26113:12710

GCTTGTTGAGCAAGAGTAGCCCAACTCATTGGTTGTGATG

>TPNB500593:305:HWWHGAFX3:1:21205:17047:2764

TCTCTTCCAAACCCAGGACCAACAACAACAATATCAATTC

>TPNB500593:305:HWWHGAFX3:1:11301:4986:8107

CCATGATACAAATGATTGTCAAACTCAACCCAAAGAAGGA

>TPNB500593:305:HWWHGAFX3:1:11302:20047:3891

TCTCTGCGCTTTCGTAGTGACGCAGCCAGTTTCTCACCGG

>TPNB500593:305:HWWHGAFX3:1:21107:5190:2515

GGCAGAGCAATTAGTTAGAGGAGAAGATATCCATCATACA

>TPNB500593:305:HWWHGAFX3:1:11107:17080:11228

CAACTTGCCTACCAATCCTTTTAGCTTTAAGAAAATCACG

>TPNB500593:305:HWWHGAFX3:1:11104:6242:12020

ATCTTATTCATCCTACAGTTACTATCCACAACAATCCTAT

>TPNB500593:305:HWWHGAFX3:2:11204:15849:14042

AAAGCATGTTGAAATTGAGGATGGTTCATCGGATGAATCC

>TPNB500593:305:HWWHGAFX3:1:11211:16529:13424

GTTCTACACCAAACACTTCAAGCCCTTCAAATCATGGACC

>TPNB500593:305:HWWHGAFX3:4:11609:17371:12511

AATAAATCGCCGTCAAGACAATTGCCCACAACCATCTCTG

>TPNB500593:305:HWWHGAFX3:1:11104:24249:18425

GATTAACGCAAAACGGTTGTTGATTGTTATTGGTGGATCT

>TPNB500593:305:HWWHGAFX3:2:11304:6085:19737

CGTTTACTCTCCTCTAACATTCTTTCACGAGCTTTTCTTT

>TPNB500593:305:HWWHGAFX3:1:21106:18293:8419

ACGGGACCAAGTCTTATAACTCTTTCATCCGGGTGTGTCA

>TPNB500593:305:HWWHGAFX3:1:11304:14103:4706

CATGCTGCTTCCAATGTAGTCATTGGCGTAACCTCCATTG

>TPNB500593:305:HWWHGAFX3:3:11510:3706:20369

GAGAGGAGCACTTTGTGACTGCAGTTGATTAGTAGGCGTG

>TPNB500593:305:HWWHGAFX3:1:11106:8302:15598

CTGCAGGAAAGCCACCTTCGACTAGACAAATTTACCAATC

>TPNB500593:305:HWWHGAFX3:1:11303:25159:3353

CAACTGAATAATCAAGAACATTAAATGGTGAAGTATATCC

>TPNB500593:305:HWWHGAFX3:3:21503:2249:15926

ATTGACACAATTTTCCCCTGATGGTGAACTTCAACCATTC

>TPNB500593:305:HWWHGAFX3:1:11207:1679:17122

TTCCAATATTTGCTTCTCATTCAATGTTCGTTGGTAATTC

>TPNB500593:305:HWWHGAFX3:4:11606:23822:10062

GAAATACCAACGAACATTGAATGAGAAGCAAATATTGGAA

>TPNB500593:305:HWWHGAFX3:1:11109:11169:2005

CGGTATTACTATCACTGGTCAAGGTAACGGTCCAATTTCT

>TPNB500593:305:HWWHGAFX3:2:21303:22351:13266

GAAGTATAATATCATAAACTGTTGTCAATTCTTTGAACAC

>TPNB500593:305:HWWHGAFX3:2:21202:18465:16129

GTTCACACATTATTTGACGAGCATGCTTTCCCATCAGGCG

>TPNB500593:305:HWWHGAFX3:2:11104:15726:12657

CTTTATAATAGGCCAATTGTAATTTTAATTTCAATTTAGT

>TPNB500593:305:HWWHGAFX3:3:11503:5590:14326

GGATTGGGTTTGTCCATGACGAATCTTCTTGGTGGAATAG

>TPNB500593:305:HWWHGAFX3:1:11109:23891:14032

ACAAATCCAAATAGGCACCGAAGACGATGTATCTGAATAG

>TPNB500593:305:HWWHGAFX3:1:21204:7097:12328

AGAATAAAAGGGGTGCCAATATGTTGGTTACTCTATGTAT

>TPNB500593:305:HWWHGAFX3:1:11202:20023:4445

ATATTTGGTCAACGGAAGGGTTTTGTTGCCATCTAATTCC

>TPNB500593:305:HWWHGAFX3:1:11208:16785:14839

CATTCCCAGCCAATCAGCTTGTGCAAGAACACCTTGGTGT

>TPNB500593:305:HWWHGAFX3:1:11206:15175:1534

GAGACACTCCAAGGCCAAAATCAATGCACCCCATGTTAAG

>TPNB500593:305:HWWHGAFX3:1:11101:25013:1311

TTTTGAAGGCTTTGATTACGATATCTGCAAACTCCACAAC

>TPNB500593:305:HWWHGAFX3:1:11208:9676:9950

CATCAGAACCATCGCTATCAAAATTTTTGTTGGGTCCATT

>TPNB500593:305:HWWHGAFX3:4:21403:6135:9558

ATGAATCTGATGTATTCCATTCTGACAAATCGTATATAAC

>TPNB500593:305:HWWHGAFX3:1:21302:16155:13142

TTTTCAGAGAGAGCCAAATCATCCCTTGATGATTCCCCAT

>TPNB500593:305:HWWHGAFX3:1:11106:15878:17521

TCAGATCGTTTTGTTTAGAAGAACAACCTATCGTTCCTCC

>TPNB500593:305:HWWHGAFX3:1:11102:23815:10449

TGAATTATCCACTATTTTGCAGACTCTTGAAGGAATGTTA

>TPNB500593:305:HWWHGAFX3:3:11601:18606:5496

TTTAATATCTTCAAGGATTTCCTTTATTGCCGCAACTAAT

>TPNB500593:305:HWWHGAFX3:1:21110:24963:5536

GGTGGCAAGTTGCTTGTATCGATAGTGGAAGCACTAGCAG

>TPNB500593:305:HWWHGAFX3:4:21507:18312:12627

CATATATACAAGATCGGGATAGTCATGACAAGCACGCTTA

>TPNB500593:305:HWWHGAFX3:1:11106:4090:13848

TTGGAGTAGCTACCAGTGATGGAAAGAGCACCTTTAACAG

>TPNB500593:305:HWWHGAFX3:1:21302:24678:10572

CGGCATAAGTGTGCACTACTTGATAAACATCTCTCGAGAC

>TPNB500593:305:HWWHGAFX3:1:11212:16641:9849

GTTGGATTTACCGGTGTGTTGTCTATATATACCGGTTCTT

>TPNB500593:305:HWWHGAFX3:3:11606:4532:9351

GTGTAACCTGGACCAGTGATGGTTTCAGTGGTCTTTTCGG

>TPNB500593:305:HWWHGAFX3:1:11104:17135:5143

GACTACCACCAACAGTGACGGTTCTGTTGAAACTGATTCT

>TPNB500593:305:HWWHGAFX3:3:11602:3797:18012

ACGATGTCCTTGCAAAACACTGCTATTCAGAATAATGGAC

>TPNB500593:305:HWWHGAFX3:1:11102:13426:10418

GACATTTCAAAACATATCGGTGCTGGAATTAGTATTGGAA

>TPNB500593:305:HWWHGAFX3:1:11310:23112:11438

GTACTTTAGCTCAATTGGACGAAGCTGTTGAAAAGAATGA

>TPNB500593:305:HWWHGAFX3:1:11202:13138:9967

GTCCTTACTCTTCTCGAGTTTTTCTGACGGTTTACTTAAT
